# Supplementary material for: De novo production of protoberberine and benzophenanthridine alkaloids through metabolic engineering of yeast
Source: Nat Commun. 2024 Oct 9;15:8759. doi: 10.1038/s41467-024-53045-3 (PMC11464499; doi:10.1038/s41467-024-53045-3)
Supplement: Supplementary file 1 — Supplementary Information [file 41467_2024_53045_MOESM1_ESM.pdf]

***De novo* production of protoberberine and benzophenanthridine alkaloids through metabolic engineering of yeast**

Jiao *et al.*

Supplementary Note 1. N-terminal truncation of BBE on its expression.

We explored the effect of N-terminal truncations of BBE on its expression level, as previous report demonstrated that signal peptide truncations would generally increase the expression level of secretory proteins[1]. Based on the sequence alignment, it predicted that CyBBE contained an ER targeting signal and a vacuole sorting signal (Supplementary Figs. 13a). Thus, we constructed three variants of CyBBE: CyBBEΔ29 (deleting amino acids 2-29), removing ER targeting signal; CyBBE29Δ46 (deleting amino acids 30-46), removing vacuolar sorting signal but retaining ER signal; and CyBBEΔ46 (deleting amino acids 2-46), removing both ER signal and vacuolar sorting signal. Unexpectedly, we observed no detectable (*S*)-SCO production when three truncations were integrated into (*S*)-RET producing strain (Supplementary Figs. 13b). To better understand this, we performed high-resolution fluorescence analysis and Western blot of these truncations that expressed in yeast.

For fluorescence imaging, three plasmids, each carrying one marker fusion protein Vph1-mRuBy2 (mRuBy2 was fused to the C-terminus of vacuole protein Vph1) and one truncation-GFP fusion protein (GFP was fused to the C-terminus of each CyBBE truncation), were transformed into yeast, respectively. Vph1-mRuBy2 was used as a control to colorize the vacuole from yeast. In contrast to wild type CyBBE forming normal GFP signal in vacuole (Fig. 4b), some bright aggregates were generated in all truncation-containing yeast cells (Supplementary Fig. 13f). These aggregates indicate improper folding of truncation constructs, as similar aggregation states of some proteins by fusing GFP to their C-terminus reported before[2-4]. In addition, we fused GFP to the C-terminus of CjNCSΔ35 from module I in this study, since CjNCSΔ35 with truncating the N-terminal signal (35 amino acids) showed an improved activity on substrates. Indeed, CjNCSΔ35-GFP expressed well, and the fluorescence filled within whole cytosol homogeneously (Supplementary Fig. 13f). It primarily indicated that functional truncations express homogeneously, instead of forming aggregates. Therefore, these results implied the importance of N-terminal signal for functional expression of CyBBE.

For the Western blot experiments, we fused His-tag to the C-terminus of each truncation, which were then transformed into yeast. Supplementary Fig. 13c showed that the protein bands for both CyBBEΔ29 and CyBBEΔ46 had similar sizes, whereas wild type CyBBE and CyBBE29Δ46 were blotted with larger bands than CyBBEΔ29 and CyBBEΔ46, and the band of wild type CyBBE was further larger than that of CyBBE29Δ46. The increased size was likely caused by post modifications, such as N-linked glycosylation. Furthermore, we simulated the structure of CyBBE using AlphaFold2 (Supplementary Fig. 13d). Two glycosylation sites at N45 and N418, and a disulfide bond forming between C37 and C96 were predicted based on the alignment with published crystal structure of homolog EcBBE (PDB file: 3D2D). Considering these potential post-translational modifications, we conducted a special Western blot with the treatment of PNGaseF, capable of specifically removing the N-linked glycosylation. No difference for CyBBEΔ29 and CyBBEΔ46 was observed no matter processing with PNGaseF or not, whereas a clear size shift appeared for CyBBE29Δ46 and wild type CyBBE after processing with PNGaseF (Supplementary Fig. 13e). These results clearly suggested that CyBBE underwent trafficking and post-translational modifications, such as N-linked glycosylation. The removal of its N-terminal signals would result in the shortage of such modifications which are otherwise essential for CyBBE expression and function. Interestingly, the protein band shift for CyBBE29Δ46 was smaller than that of wild type CyBBE, possibly due to lack of N45-linked glycosylation for CyBBE29Δ46. All these results highlighted that the N-terminus of CyBBE was essential for its correct folding and proper structure formation.

Supplementary Note 2. Potential mechanisms of ER retrograding strategy for improved BBE activity.

To better understand possible mechanisms of ER retrograding strategy, we performed RT-qPCR, Western blot, *in vitro* activity assays with various pH, as well as molecular dynamic simulations to compare the difference between CyBBE_ERTS and wild type CyBBE. In summary, our ER targeting strategy could maintain proper N-terminal signal thus allowing for CyBBE guided into secretory pathway for processing and post-modifications, while at the same time the strategy could direct CyBBE back to ER, which provides a more favorable microenvironment for its optimal function, instead of its natural destination vacuole.

Inspired by glycosylation on wild type CyBBE, we conducted Western blot with the treatment of PNGaseF for both CyBBE_ERTS and GOTS_CyBBE. Indeed, both variants showed similar band shift compared with wild type CyBBE after PNGaseF treatment (Supplementary Fig. 15d), suggesting that they have gone through similar post-modifications. Quantitative PCR and Western blot showed similar trends between transcription and protein expression level for these constructs. The transcription level and expression level of Golgi targeted protein GOTS_CyBBE were significantly increased, while ER targeted protein CyBBE_ERTS showed comparable levels relative to wild type CyBBE (Supplementary Figs. 15b and 15c). Thus, an over 2-fold increase in the conversion of (*S*)-RET to (*S*)-SCO for CyBBE_ERTS would not be explained by enzyme expression level (Supplementary Figs. 15a, 15b and 15c).

Besides proper expression and correct folding, different microenvironments exhibited remarkable differences, such as pH, which is around 5.6 in vacuole while for ER it is around 7.0[5]. To evaluate the effect of different pH on its activity, we assessed *in vitro* activity of CyBBE_ERTS and CyBBE under different pHs within Citrate-phosphate buffer[6] (pH from 4.7 to 8.0) or tris-HCl buffer (pH 7.5 and pH 8.6). Strikingly, the conversion efficiency was gradually increasing along with the pH increase for both CyBBE_ERTS and CyBBE (Supplementary Figs. 16a and 16b). Also, a trend of decrease on the substrate (*S*)-RET was observed for both CyBBE_ERTS and CyBBE (Supplementary Fig. 16c). This result was consistent with previous study that BBE displayed an optimal pH at 8.9 *in vitro*[7]. The catalytic mechanism of homolog EcBBE (*Eschscholzia californica*) has been investigated in detail and has been shown that Glu417 (Glu425 in CyBBE) plays a crucial role as catalytic base necessary for carbon-carbon bond formation[8, 9]. The deprotonated side chain of Glu417 acts to deprotonate the phenolic C3'-OH group, thereby increasing the nucleophilicity of the C2' carbon and facilitating an S_N2-type attack onto the N-methyl group of the substrate (*S*)-RET to form carbon-carbon bond in a Friedel-Crafts–like alkylation reaction (Supplementary Fig. 16d). This was supported by the Glu417Gln mutant, which displays a 1500-fold reduction on catalytic efficiency[8]. Glu has three pKa values at 2.10, 4.97, 9.47. While it is challenging to determine the exact pKa value of the side chain of Glu417 within the protein environment, the carboxylic group of Glu417 is more likely to be deprotonated in alkaline conditions above its pKa as predicted (https://playground.calculators.cxn.io/) (Supplementary Fig. 16e). In addition, the charge of the amine group in the substrate (*S*)-RET varies significantly along with rising pH, changing from 98% positively charged at pH 6 to 5% charged at pH 9 (https://playground.calculators.cxn.io/) (Supplementary Fig. 16f). A decrease in the N-positive charge results in higher electron density around the amine group, thereby increasing the likelihood of hydride removal from the N-methyl group to FAD (Supplementary Fig. 16d). This hydride transfer (C-H bond cleavage) was proposed to be rate-limiting during flavin reduction, which requires the amine group of substrate retaining uncharged state for maximal activity[10]. Furthermore, the intermediate substance requires to be further deprotonated to form the 'berberine bridge', which could be favored under alkaline conditions (Supplementary Fig. 16d). Considering these mechanistic insights, an acidic environment would be unfavorable for the BBE-catalyzed reaction. Since yeast vacuole is more acidic compared to the more neutral environment of the ER, the pH effect elucidates the catalytic mechanism of ER targeting strategy for improved BBE activity, which is consistent with our *in vitro* assay results (Supplementary Figs. 16a, 16b and 16c). Moreover, besides (*S*)-SCO, 13,14-dihydroscoulerine and dehydroscoulerine were also produced when pH was above 6.0 (Supplementary Figs. 16a and 16b). These results agreed well with a prior report that BBE could catalyze the six electrons oxidation of (*S*)-RET to dehydroscoulerine[11]. Although this experiment was not perfect to mimic *in vivo* conditions as

seen the differences by using different buffers, it highlighted the importance of subcellular environment for achieving optimal activity.

Furthermore, molecular dynamic simulation was conducted to explore the difference between CyBBE_ERTS (CyBBE-GSGSHDEL) and wild type CyBBE. For this purpose, one additional construct CyBBE-GSGSVIML was designed as a negative control since the C-terminal tail VIML consisted of hydrophobic amino acids without hydrogen bond acceptors and donors. To test the conversion efficiency of these three candidates, these constructs were integrated into (*S*)-RET producing platform strain XJ0691. We found that CyBBE-GSGSHDEL led to over 2-fold improvement of product (*S*)-SCO, whereas CyBBE-GSGSVIML resulted in modest decrease compared to wild type CyBBE (Supplementary Fig. 17a). To understand the effects of C-terminal modifications, their structures were first predicted by Alphafold2. By using molecular docking and dynamic simulations, we observed that the structure of CyBBE-GSGSHDEL exerted a higher degree of convergence under the function of the tail HDEL during 50 ns simulations, while CyBBE and CyBBE-GSGSVIML tended to be of incompactness (Supplementary Fig. 17b). Such value of convergence could be also reflected in several other ways, including RMSD (Root Mean Square Deviation), Rg (Radius of Gyrate) and SASA (Solvent-access surface area). The results indeed showed that RMSD, Rg and SASA of protein CyBBE-GSGSHDEL were decreased, suggesting that such HDEL tails likely improve the compactness value of protein structure, whereas these parameters in terms of protein CyBBE-GSGSVIML exhibited increased trend (Supplementary Figs. 17c, 17d and 17e). Moreover, the molecular dynamic simulation indicated that the C-terminal tail HDEL interacts with the enzyme BBE itself, and two extra hydrogen bonds might be formed when the tail HDEL fused to the C-terminus of CyBBE (Lys87-Asp554 and Gln88-Glu555) (Supplementary Fig. 17f). Two additional malfunctional ER tails KDEL and RDEL were also tested, and the corresponding strains produced higher (*S*)-SCO production with only around 60% than that expressing wild type CyBBE (Supplementary Fig. 17a). These results inferred that the C-terminus HDEL not only improved the stability of CyBBE, but also guaranteed the protein retrograding to ER, where a more favorable microenvironment is provided. Such synergy resulted in the increase of CyBBE activity, responsible for over 2-folds conversion from substrate (*S*)-RET to (*S*)-SCO.

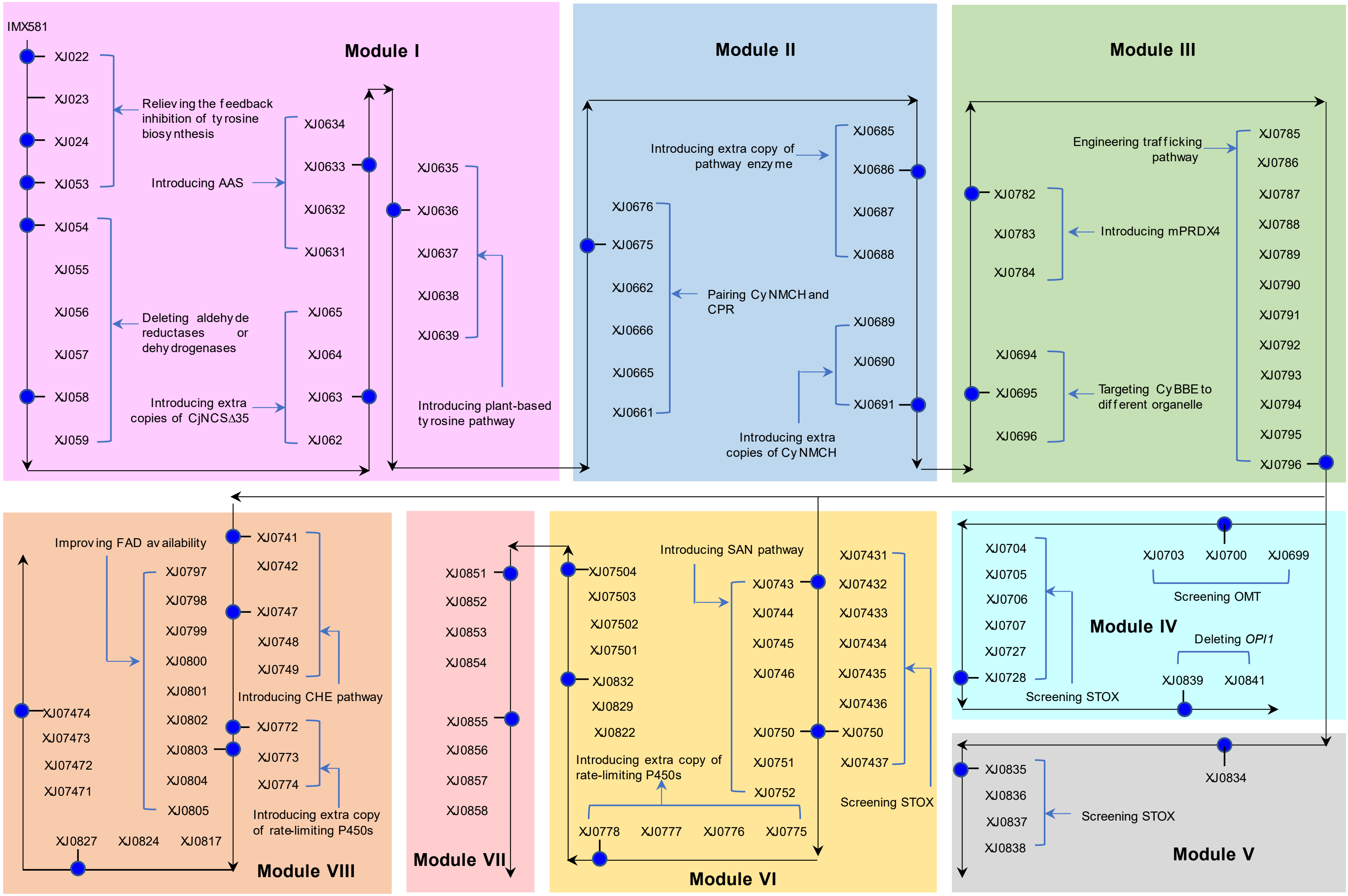

Supplementary Figure 1. Construction flowchart of engineered strains in this study.

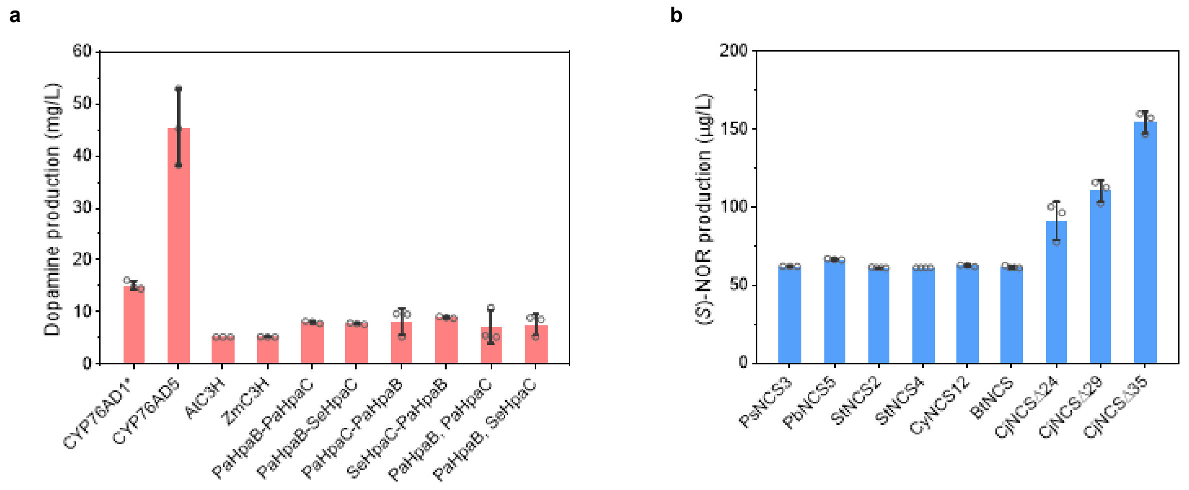

Supplementary Figure 2. Screening the optimal candidate for pathway reconstruction in module I. a Screening the optimal tyrosine hydrolase by measuring the dopamine titers in strains (XJ001 and XJ011-XJ019) co-expressing DODC. HpaC, acting as the redox partner, enables to synthesize cofactor FADH2 from NAD(P)H, and then transfers the cofactor to HpaB enzyme for the hydroxylation[12]. Both enzymes were co-expressed separately or fused together to generate diverse chimeras (PaHpaB-PaHpaC, PaHpaB-SeHpaC, PaHpaC-PaHpaB and SeHpaC-PaHpaB) with a GGSGGS linker for expression, aiming to improve the electron transfer efficiency. **b** Screening the optimal norcoclaurine synthase by measuring the (*S*)-NOR titers in strains (XJ002-XJ010) carrying CYP76AD1* and DODC. Significance was calculated using two-tailed *t*-test. Data are presented as mean ± standard deviations (n = 3 or 4 biologically independent samples). Source data are provided as a Source Data file.

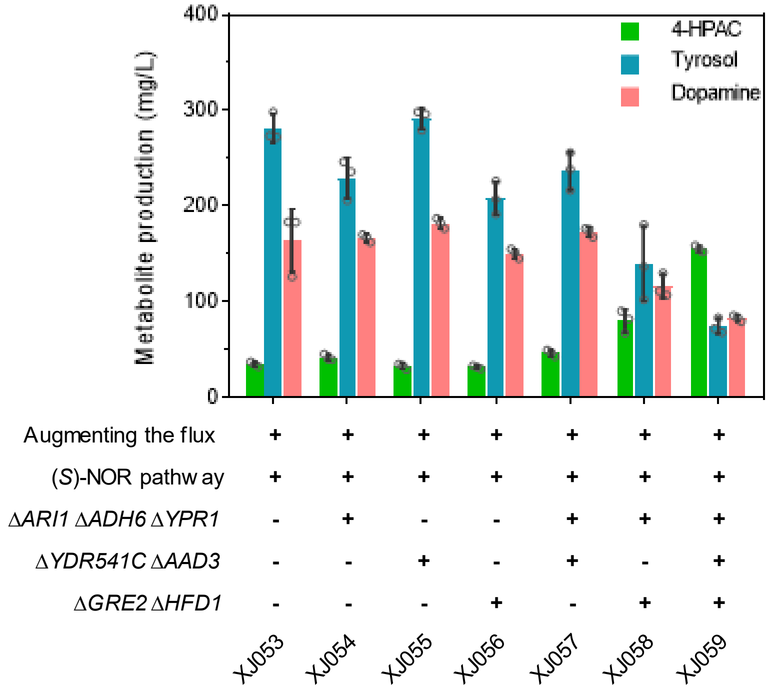

Supplementary Figure 3. Dopamine, by-products 4-HPAC and tyrosol formation in engineered strains. By-products production to some extent could reflect that the conversion of 4-HPAA towards (*S*)-NOR biosynthesis was limited. Augmenting the flux indicates overexpression of yeast native *ARO1*, *ARO2*, *ARO3*, and expression of EcAROL, MtPDH1, ARO4^{K229L} and ARO7^{G141S}. (*S*)-NOR pathway indicates the introduction of optimal CYP76AD5, DODC and CjNCSΔ35. Significance was calculated using two-tailed *t*-test. Data are presented as mean ± standard deviations (n = 3 biologically independent samples). Source data are provided as a Source Data file.

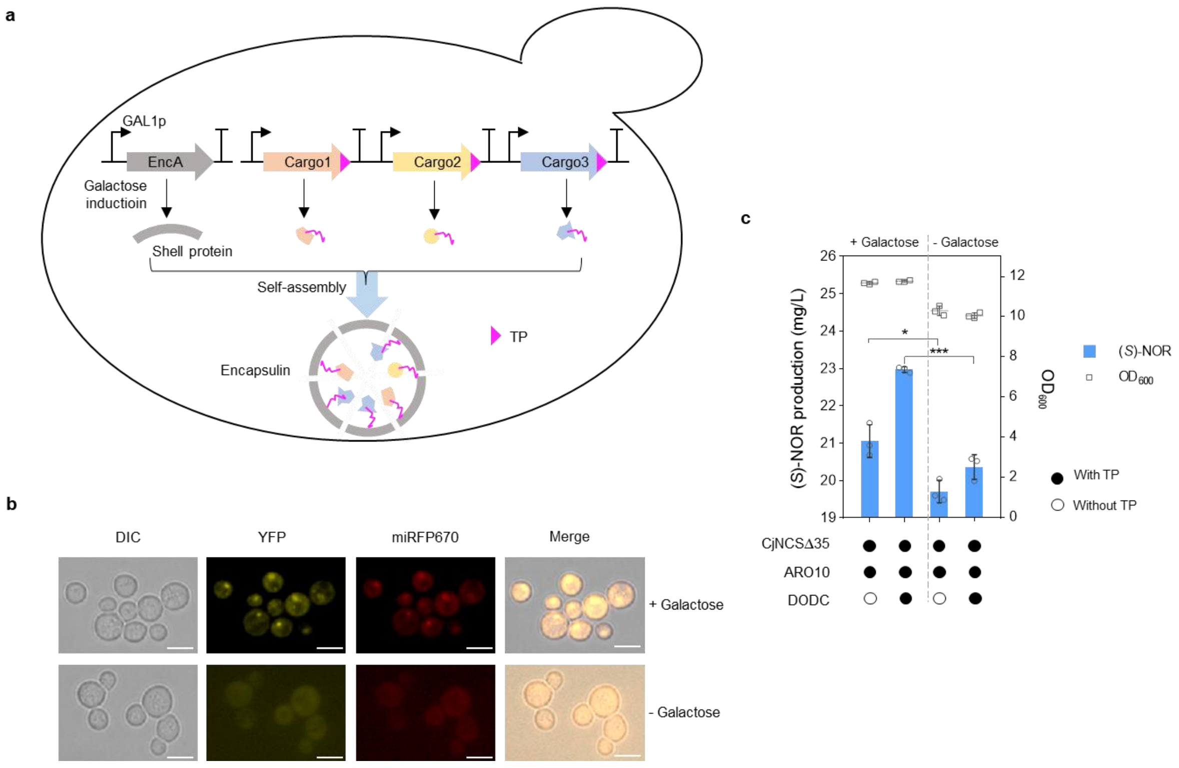

Supplementary Figure 4. Cargo proteins encapsulation in yeast. a Schematic illustration of the compartmentation of cargo proteins during encapsulin self-assembly process. Shell protein encoding gene EncA was expressed under the control of GAL1 promoter. Heterologous cargo proteins were fused with a C-terminal targeting peptide (TP, LTVGSLRR). **b** Fluorescence microscope images of yeast cells expressing EncA and three cargos, including V_N-TP, V_C-TP and miRFP670-TP, under galactose induction. To test if multiple heterologous proteins can be co-localized inside encapsuling compartments, we used a developed split-venus strategy, in which an elevated intensity of fluorescence was observed only when two splitted venus fragments (V_N and V_C) were brought into proximity in spatial for self-assembly[13]. The shell gene EncA was expressed under the control of inducible GAL1 promoter in IMX581, then two splitted parts V_N, V_C and a complete red fluorescent protein (miRFP670) fused with C-terminal TP, were incorporated. Scale bar represents 10 μm. **c** (*S*)-NOR titers and final OD_{600} in engineered strains in which capsid EncA was controlled by GAL1p, along with CjNCSΔ35-TP and ARO10-TP were expressed (XJ028), or CjNCSΔ35-TP, ARO10-TP and DODC-TP were expressed (XJ029) with or without galactose induction. Solid circle represents cargo expression fused with TP; empty circle represents cargo expression without TP. Cells were cultured for 72 h in 20 mL delft media with 20 g/L glucose and 10 g/L galactose as inducer. Significance was calculated using two-tailed *t*-test. Data are presented as mean ± standard deviations (n = 3 biologically independent samples). Source data are provided as a Source Data file.

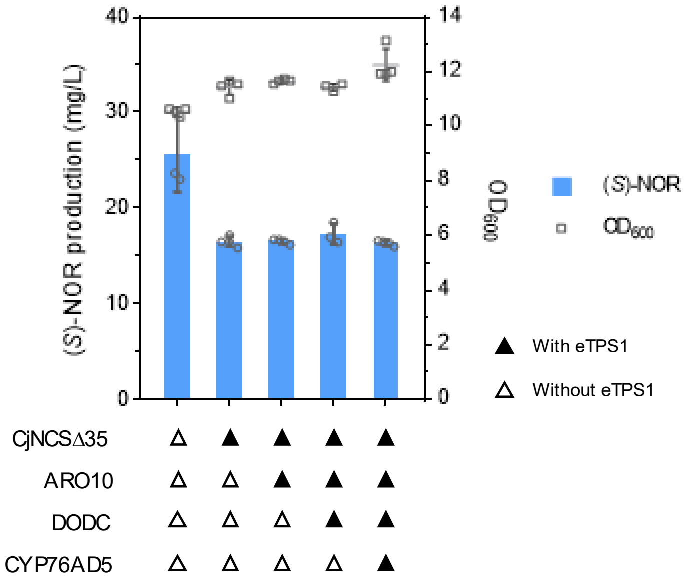

Supplementary Figure 5. Peroxisome compartmentalization of (*S*)-NOR biosynthesis-related genes in yeast. (*S*)-NOR titers and final OD_{600} in engineered strains (XJ053, XJ0234-XJ0237) in which CjNCSΔ35 was targeted to the peroxisome lumen by fusing an engineered C-terminal enhanced PTS1 (ePTS1), and other three upstream enzymes *ARO10*, DODC and CYP76AD5 were sequentially fused with ePTS1. Solid triangle represents cargos expression fusing with ePTS1; empty triangle represents cargos expression without ePTS1. Significance was calculated using two-tailed *t*-test. Data are presented as mean ± standard deviations (n = 3 or 4 biologically independent samples). Source data are provided as a Source Data file.

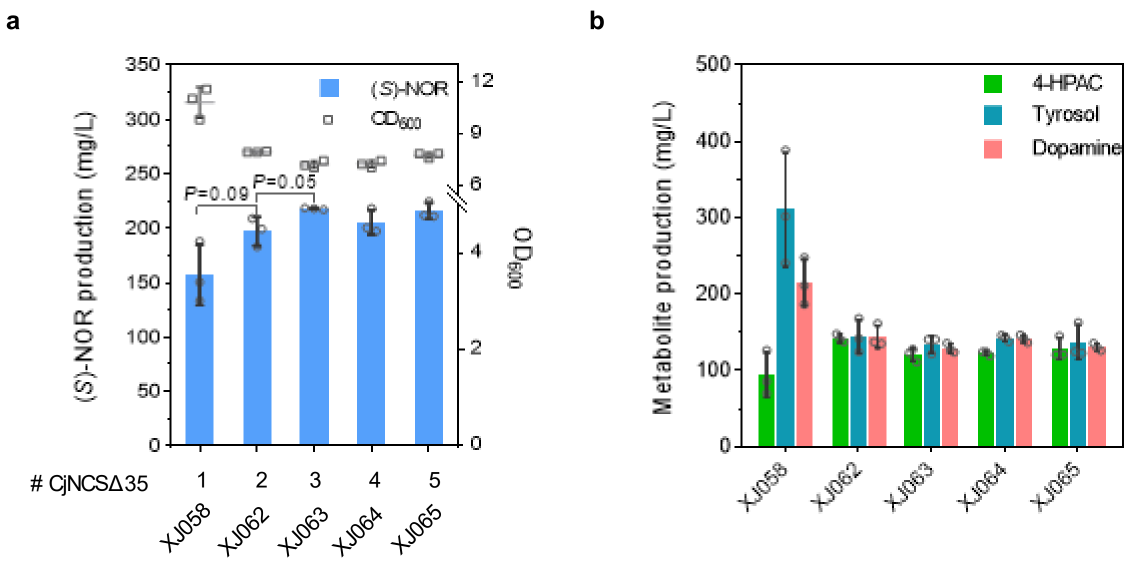

Supplementary Figure 6. The introduction of additional copies of rate-limiting enzyme CjNCSΔ35 to increase the (*S*)-NOR titer in yeast. a (*S*)-NOR titers and final OD_{600}, **b** dopamine, by-products 4-HPAC and tyrosol titers in engineered strains containing more copies of CjNCSΔ35. Data are presented as mean ± standard deviations (n = 3 biologically independent samples). Source data are provided as a Source Data file.

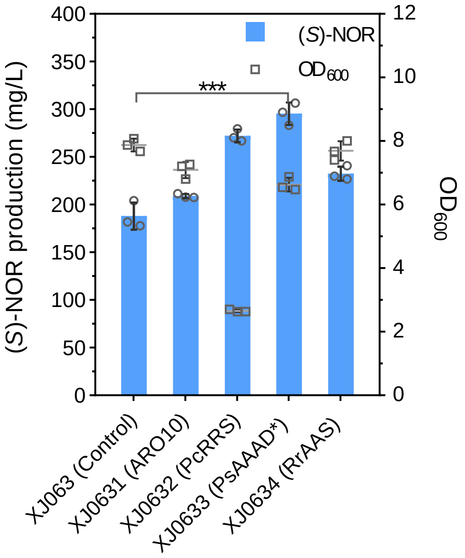

Supplementary Figure 7. Increasing the availability of 4-HPAA to improve the (*S*)-NOR titer in yeast. (*S*)-NOR titers and final OD_{600} in engineered strains containing various AAS candidates or extra copy of *ARO10*. AAS could catalyze tyrosine to generate 4-HPAA directly, while *ARO10* enables decarboxylation of phenylpyruvate (HPP) to produce 4-HPAA. Data are presented as mean ± standard deviations (n = 3 biologically independent samples). Source data are provided as a Source Data file.

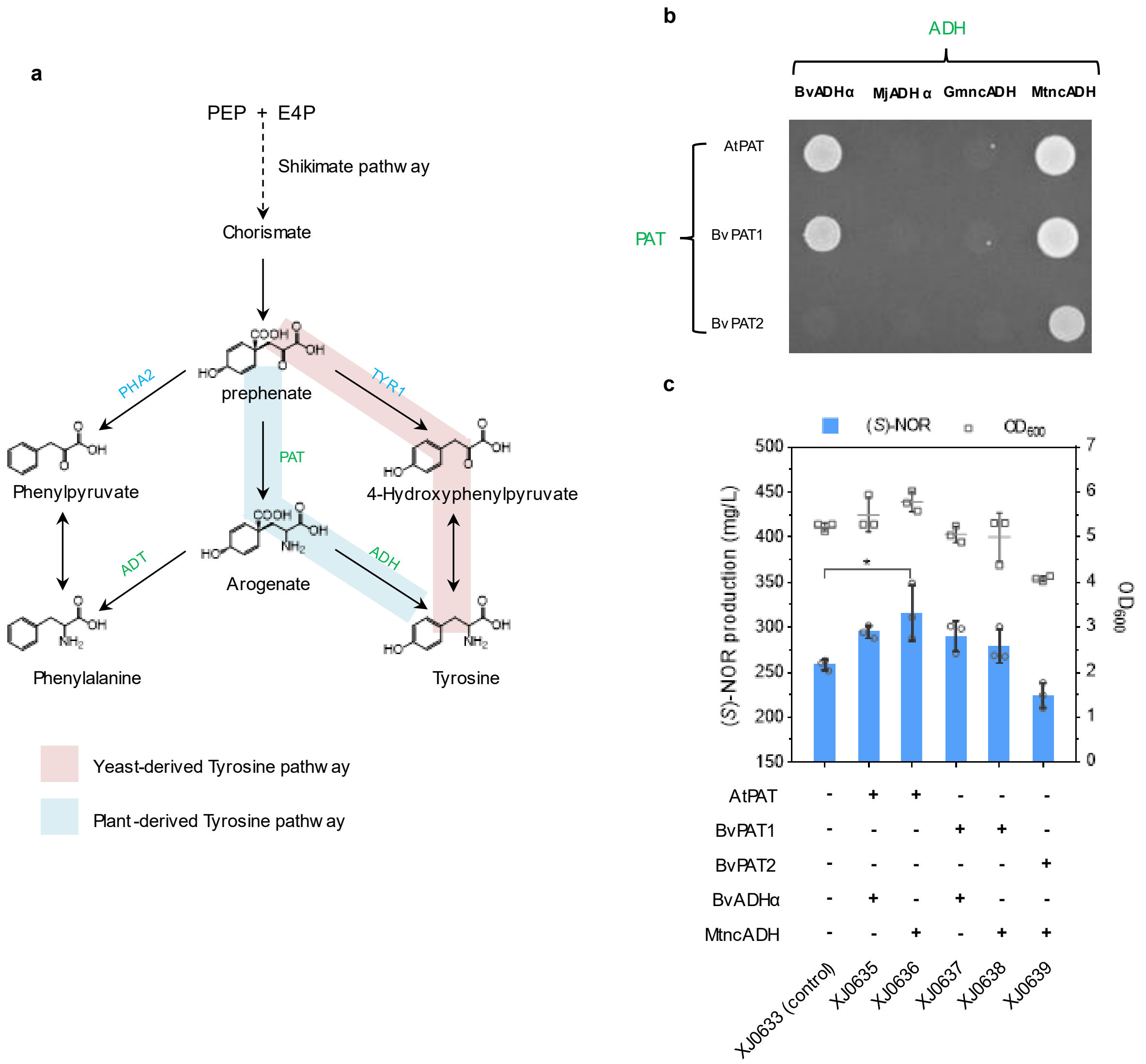

Supplementary Figure 8. The introduction of plant-based tyrosine biosynthetic pathway to improve the (*S*)-NOR titer in yeast. a Schematic presentation of tyrosine and phenylalanine biosynthesis in yeast and plant. Generally, prephenate is first subjected to dehydration and decarboxylation by prephenate dehydrogenase to produce 4-hydroxyphenylpyruvate (4-HPP), then transamination to biosynthesize tyrosine in yeast. Alternatively in most plants, these reactions reversely occur, with first transamination to arogenate by PPA aminotransferase (PAT), then dehydrogenation and decarboxylation by arogenate dehydrogenase (ADH) to generate tyrosine[14]. **b** Growth images of *TYR1*-deficiency strain IMX581 incorporating plant-derived tyrosine pathway. 10 μL preculture was plated on SD plate without tyrosine addition at 30°C for 3 days. **c** (*S*)-NOR titers and final OD_{600} in engineered strains containing functional enzyme combination of PAT and ADH. Data are presented as mean ± standard deviations (n = 3 biologically independent samples). Source data are provided as a Source Data file.

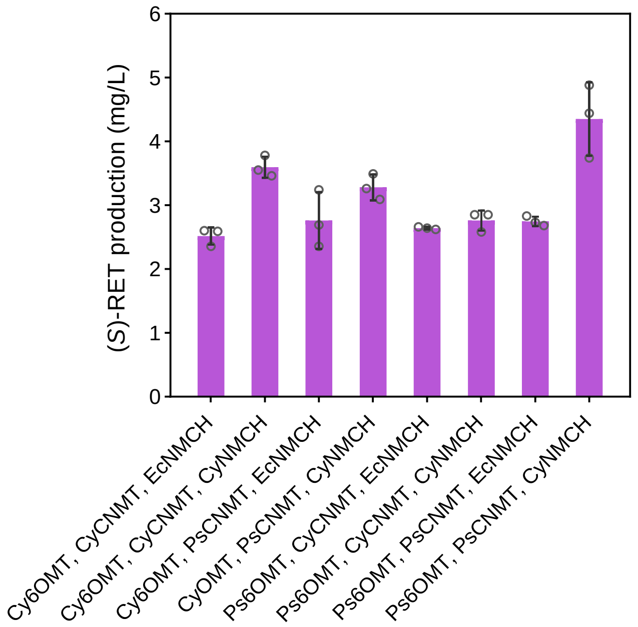

Supplementary Figure 9. Screening the optimal enzyme combination of first three steps in module II to biosynthesize (*S*)-RET. Eight combinations were integrated into genetically background-simple strain XJ040, harboring PsCPR and Ps4'OMT to implement (*S*)-RET biosynthesis. Data are presented as mean ± standard deviations (n = 3 biologically independent samples). Source data are provided as a Source Data file.

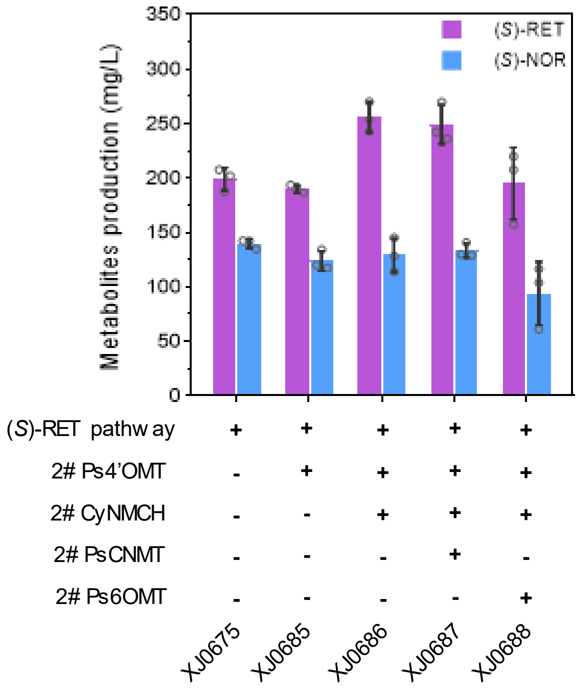

Supplementary Figure 10. (*S*)-RET and (*S*)-NOR titers in strains containing extra copy of enzymes from module II. (*S*)-RET pathway indicates the introduction of Ps6OMT, PsCNMT, CyNMCH, ATR1 and Ps4'OMT. Data are presented as mean ± standard deviations (n = 3 biologically independent samples). Source data are provided as a Source Data file.

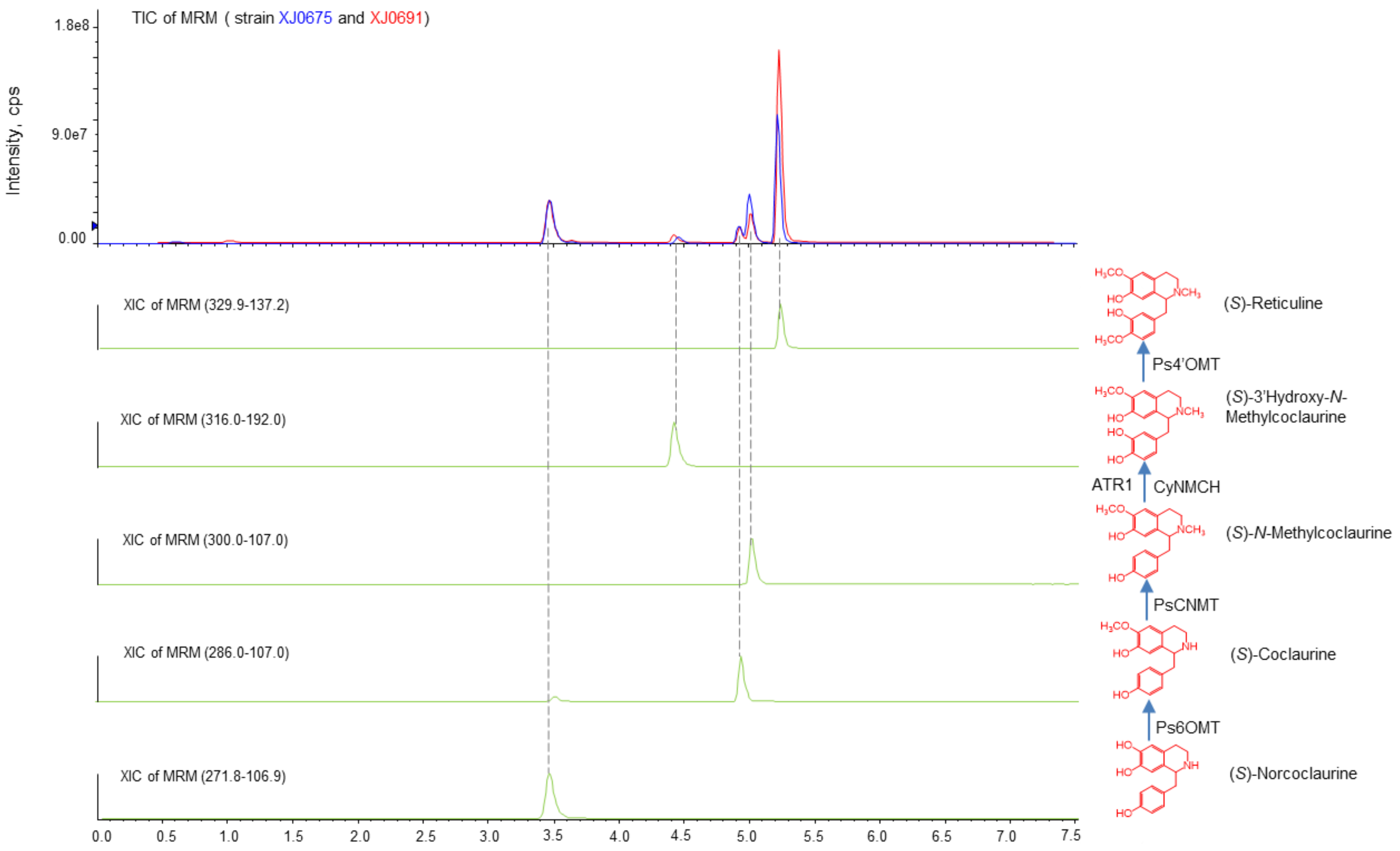

Supplementary Figure 11. TIC of module II intermediates in strain XJ0691 and XJ0675 by LC-MS/MS analysis with MRM transition mode. Blue indicates strain XJ0675 where the (*S*)-RET biosynthesis pathway was reconstructed; red indicates strain XJ0691 which was derived from strain XJ0675, carrying two copies of Ps4'OMT and five copies of CyNMCH. Three intermediates (*S*)-soclaurine, (*S*)-*N*-methylcoclaurine, 3'hydroxy-*N*-methylcoclaurine were detected by MRM transitions based on previously published spectra parameters (286.0-107.0, 300.0-107.7, 316.0-192.0)[15], while (*S*)-NOR and (*S*)-RET were measured by MRM transitions based on authentic standards.

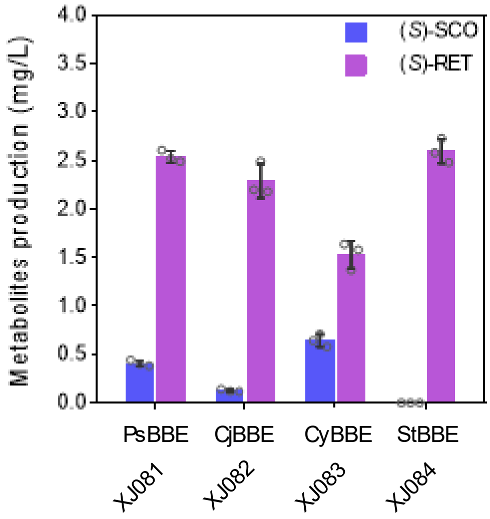

Supplementary Figure 12. Screening the optimal BBE candidate to produce (*S*)-SCO in module III. Variously originated BBE homologs were chromosomally integrated into genetically background-simple strain XJ048. Data are presented as mean ± standard deviations (n = 3 biologically independent samples). Source data are provided as a Source Data file.

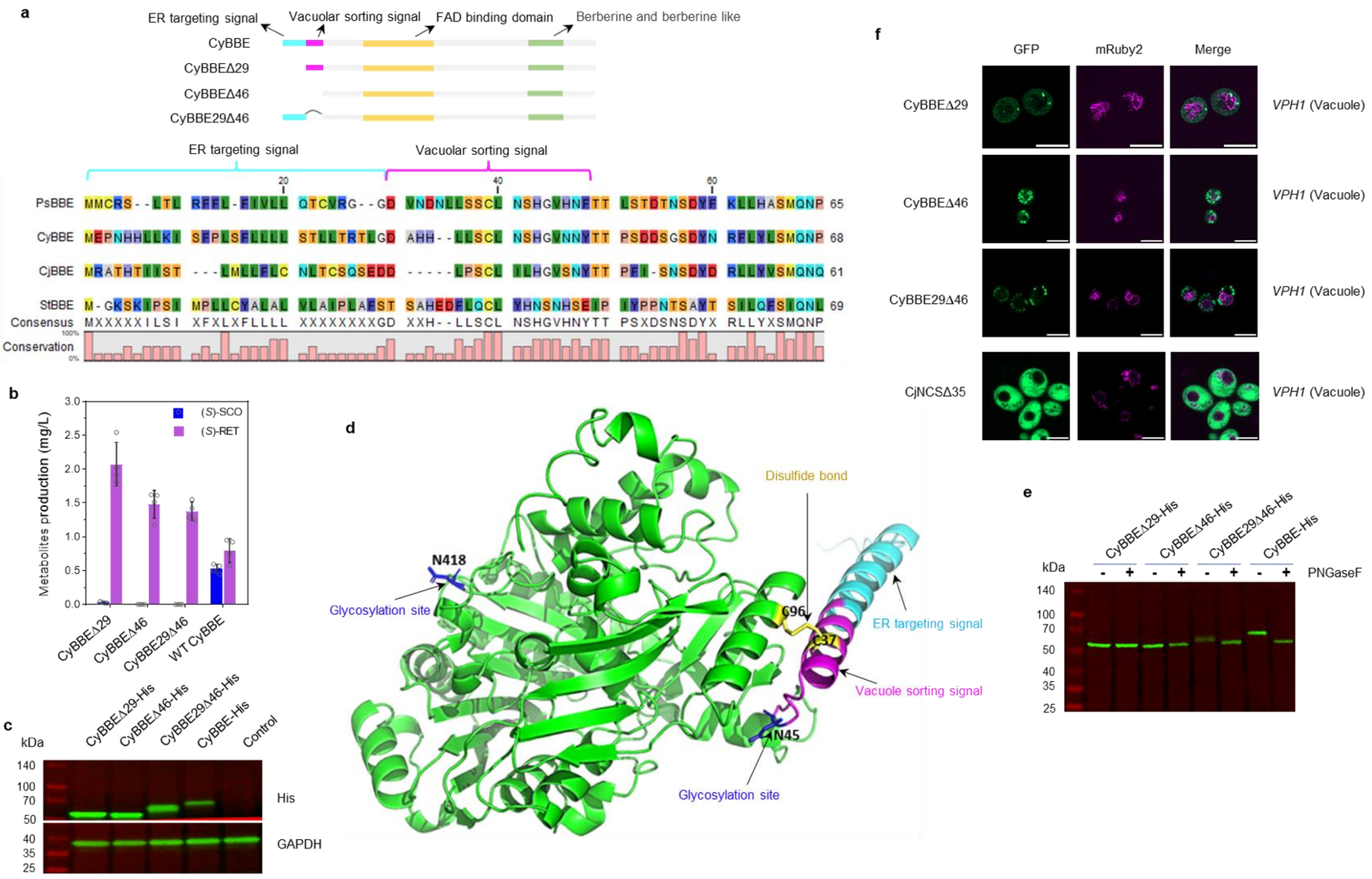

Supplementary Figure 13. Prediction and truncation of signal peptides of BBEs. a Schematic overview of protein sequence alignment of BBE candidates. BBE was characterized to contain the conserved motifs, FAD binding domain and berberine and berberine like domain, based on Pfam analysis, and ER targeting sequence and vacuolar sorting sequence according previous *in vitro* assays[16]. Sequence alignment was performed by CLC Sequence Viewer version 8. Cyan and magenta indicate predicted ER targeting peptide and vacuolar sorting determinant of CyBBE, respectively. Three truncations were illustrated in the diagram. CyBBEΔ29 (deleting amino acids 2-29), deleting ER target signal; CyBBE29Δ46 (deleting amino acids 30-46), removing vacuolar sorting signal but retaining ER signal; and CyBBEΔ46 (deleting amino acids 2-46), indicating deletion of both ER signal and vacuolar sorting signal. **b** (*S*)-SCO and (*S*)-RET titers from engineered strains expressing various CyBBE truncations. **c** Western blot analysis of CyBBE and its three truncations. Plasmids carrying CyBBE or its truncations fused with His tag, were transformed into wild type IMX581. *GAPDH* acted as a gatekeeper gene. Normalization was performed by using Image Lab software. Data are presented as mean ± standard deviations (n = 4 biologically independent samples). **d** The predicted protein structure of CyBBE by AlphaFold2. The disulfide bond was formed between Cys37 (C37) and Cys96 (C96), which are marked with yellow. The predicted glycosylation sites at Asn45 (N45) and Asn418 (N418) are represented with blue. **e** Western blot analysis of CyBBE and three truncations under PNGaseF treatment for removing the potential N-glycosylation. **f** Fluorescence images of CyBBEΔ29, CyBBE29Δ46, CyBBEΔ46 and CjNCSΔ35 in yeast obtained by confocal microscope. The plasmid carrying each CyBBE truncations or CjNCSΔ35 with C-terminal GFP tag and mRuBy2-fused marker proteins Vph1 were transformed into wild type IMX581. Scale bar represents 5 µm. Source data are provided as a Source Data file.

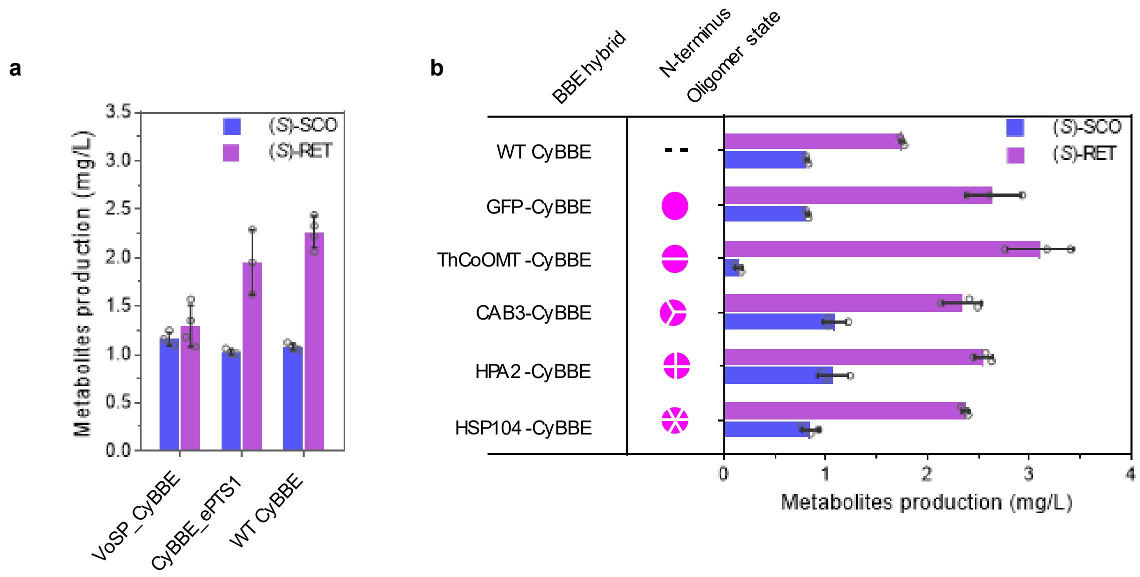

Supplementary Figure 14. Modifying CyBBE. a (*S*)-SCO and (*S*)-RET titers from engineered strains expressing wild type CyBBE or its variants with modifications of ER signal peptides replacement or fusion with a C-terminal ePTS1. Predicted signal peptides of CyBBE was substituted with a 24-amino acid vacuolar signal sequence from Proteinase A. **b** (*S*)-SCO and (*S*)-RET titers from engineered strains carrying CyBBE variants by fusion with N-terminal soluble domains displaying various oligomerization state. Pieces of pink circle indicate various oligomerization state. GFP, green fluorescence protein (monomer); TfCoOMT, heterologous columbamine *O*-methyltransferase from *T. flavum* (dimer); *CAB3*, endogenous subunits of the phosphopantothenoylcysteine decarboxylase (PPCDC) complex (trimer); *HPA2*, endogenous histone acetyltransferase (tetramer); *HSP104*, endogenous heat shock protein (hexamer). Data are presented as mean ± standard deviations (n = 3 or 4 biologically independent samples). Source data are provided as a Source Data file.

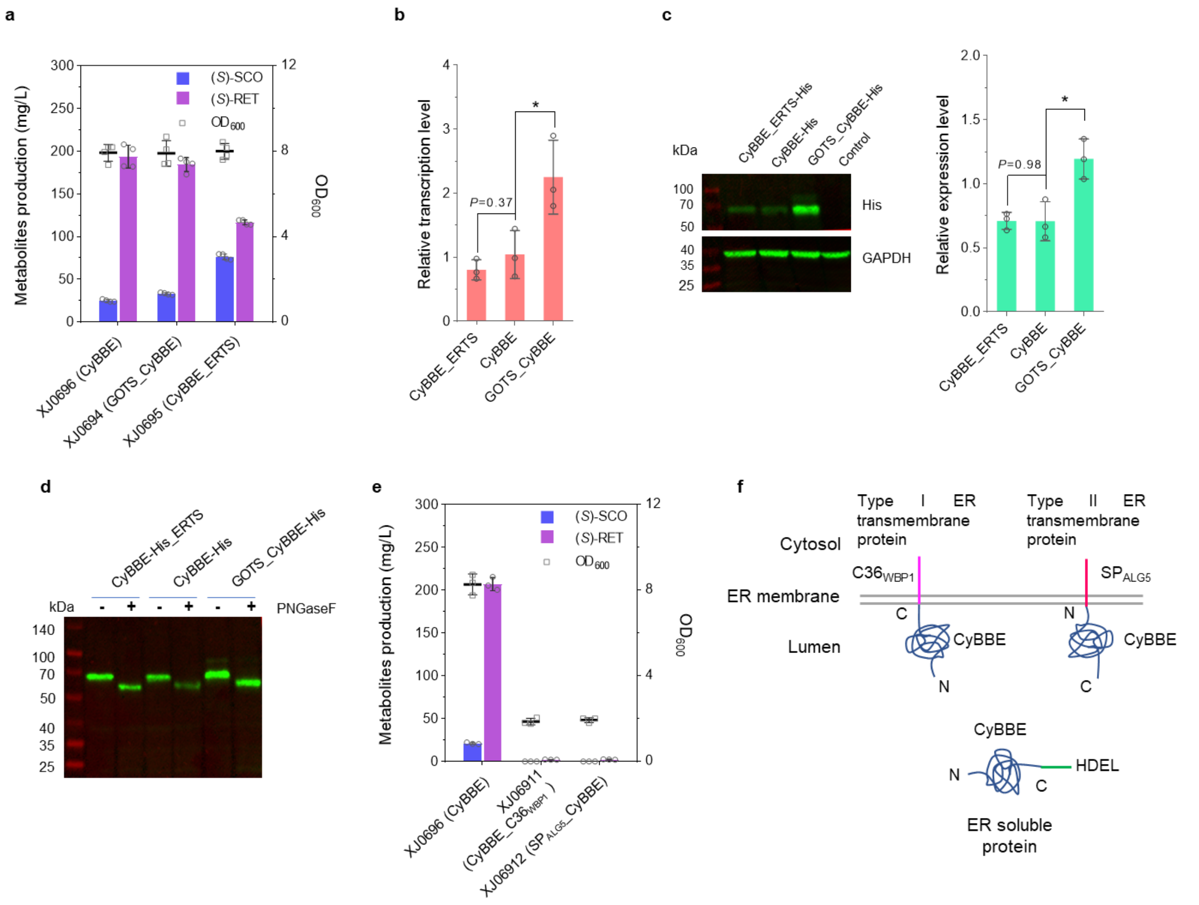

Supplementary Figure 15. Engineering CyBBE to re-localize it to ER and Golgi. a (*S*)-SCO and (*S*)-RET titers from engineered strains expressing CyBBE or its variants, CyBBE_ERTS and GOTS_CyBBE. **b** QRT-PCR analysis of CyBBE and two variants. *ACT1* acted as gatekeeper gene. **c** Western blot analysis of CyBBE and two variants. GAPDH acted as gatekeeper gene. Normalization was performed by using Image Lab software. **d** Western blot analysis of CyBBE and two variants under PNGase F treatment for removing the potential N-glycosylation. **e** (*S*)-SCO and (*S*)-RET titers from engineered strains expressing CyBBE or its variants, type I transmembrane ER protein CyBBE_$C36_{WBP1}$ and type II transmembrane ER protein SP_{ALG5}_CyBBE. **f** Schematic presentation of type I transmembrane ER protein CyBBE_$C36_{WBP1}$, type II transmembrane ER protein SP_{ALG5}_CyBBE and ER soluble protein CyBBE_ERTS. Data are presented as mean ± standard deviations (n = 3 biologically independent samples). Source data are provided as a Source Data file.

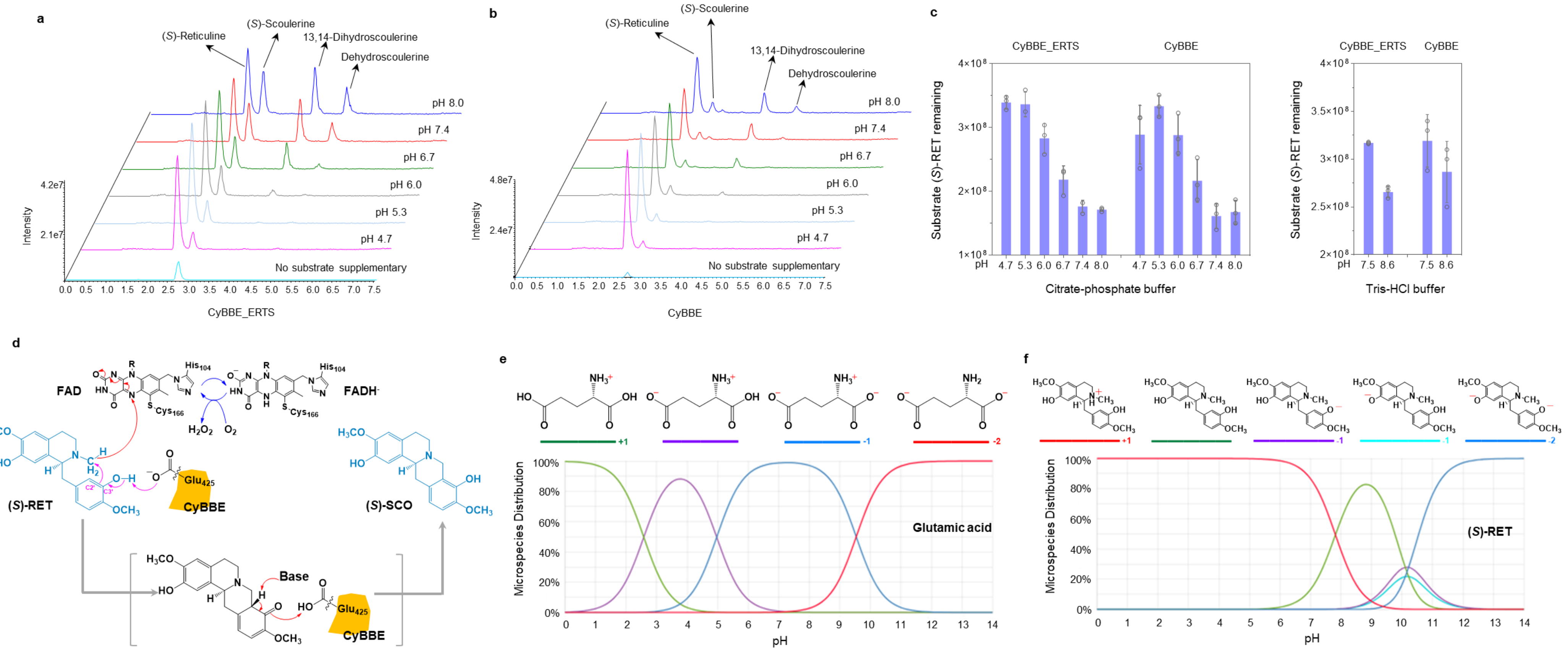

Supplementary Figure 16. ***In vitro*** **activity assays of CyBBE_ERTS and CyBBE under varied pH conditions.** LC-MS/MS analysis of **a** CyBBE_ERTS and **b** CyBBE catalyzed reactions in various citrate-phosphate buffer with different pH. **c** The consumption of susbtrate (*S*)-RET by CyBBE_ERTS or CyBBE *in vitro* catalyzation reaction within citrate-phosphate buffer[6] or tris-HCl buffer. **d** Proposed reaction mechanism for the CyBBE-catalyzed reaction. BBE catalyzes carbon–carbon bond formation between the N-methyl group and the C2' carbon of (*S*)-RET. The deprotonated side chain of Glu417 acts crucially to deprotonate the phenolic C3'-OH group, thereby increasing the nucleophilicity of the C2' carbon and facilitating an S_N2-type attack onto the N-methyl group of the substrate (*S*)-RET to form the 'berberine bridge'. The microspecies distribution of **e** glutamic acid and **f** (*S*)-RET in distinct pH environment predicted by accessible calculators (https://playground.calculators.cxn.io/). Data are presented as mean ± standard deviations (n = 3 biologically independent samples). Source data are provided as a Source Data file.

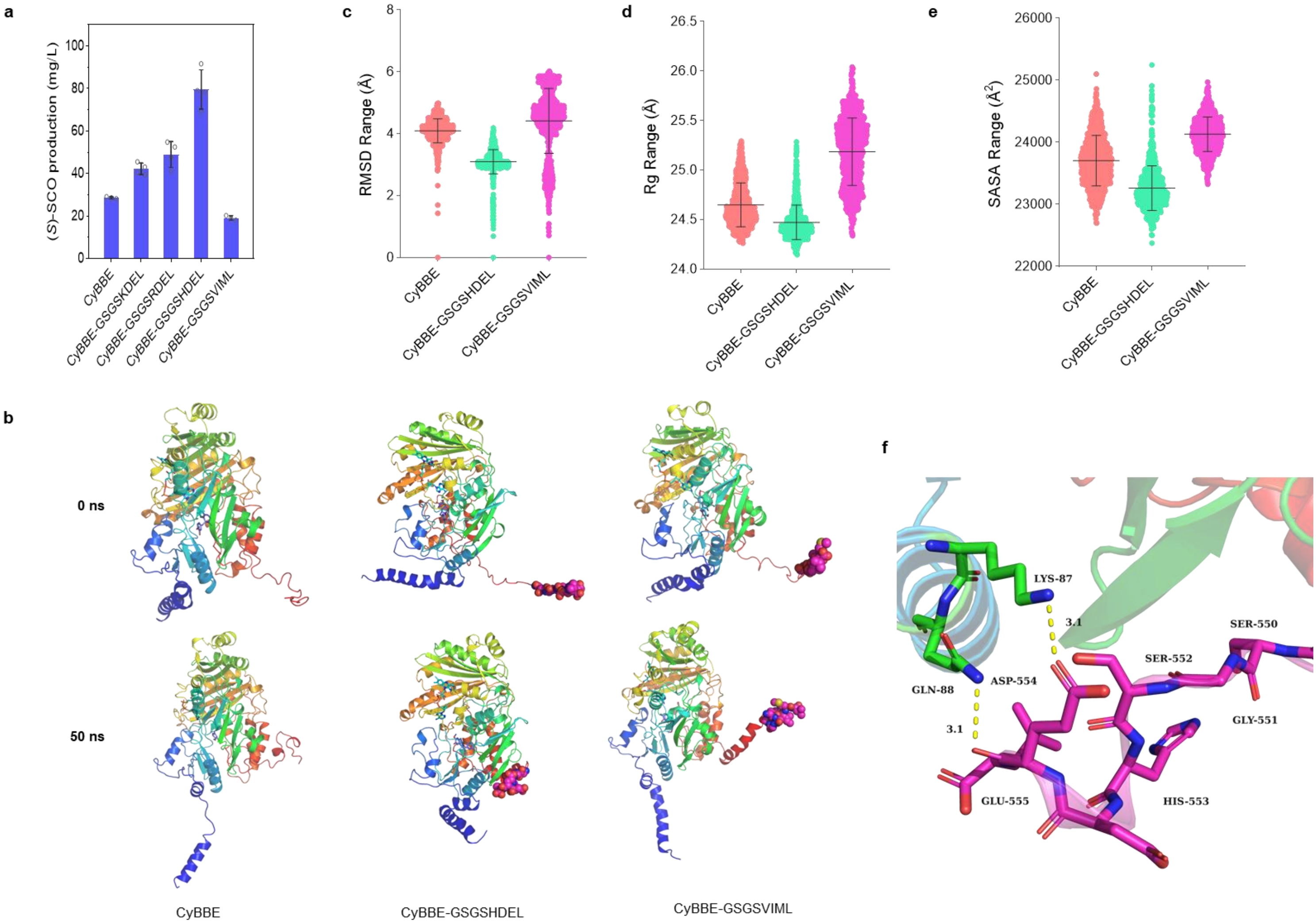

Supplementary Figure 17. Molecular dynamic simulations of CyBBE and its C-terminal tail fused variants. a The production of (*S*)-SCO in strains containing CyBBE, CyBBE-GSGSKDEL, CyBBE-GSGSRDEL CyBBE-GSGSHDEL and CyBBE-GSGSVIML, respectively. **b** The variations between the initial state and 50 ns simulation, **c** RMSD (Root Mean Square Deviation), **d** Rg (Radius of Gyrate) and **e** SASA (Solvent-access surface area) for CyBBE, CyBBE-GSGSHDEL and CyBBE-GSGSVIML. Proteins are shown with colored ribbons, and terminally modified amino acids are shown with purple spheres. The FAD cofactor is shown with a cyan stick, and the substrate is shown with a light blue stick. **f** Two hydrogen bonds might form between the HDEL tail and BBE itself (Lys87-Asp554 and Gln88-Glu555). Tail amino acids are shown with magenta sticks, and hydrogen bonds are shown with yellow dotted lines. Data are presented as mean ± standard deviations (n = 3 or 4 biologically independent samples). Source data are provided as a Source Data file.

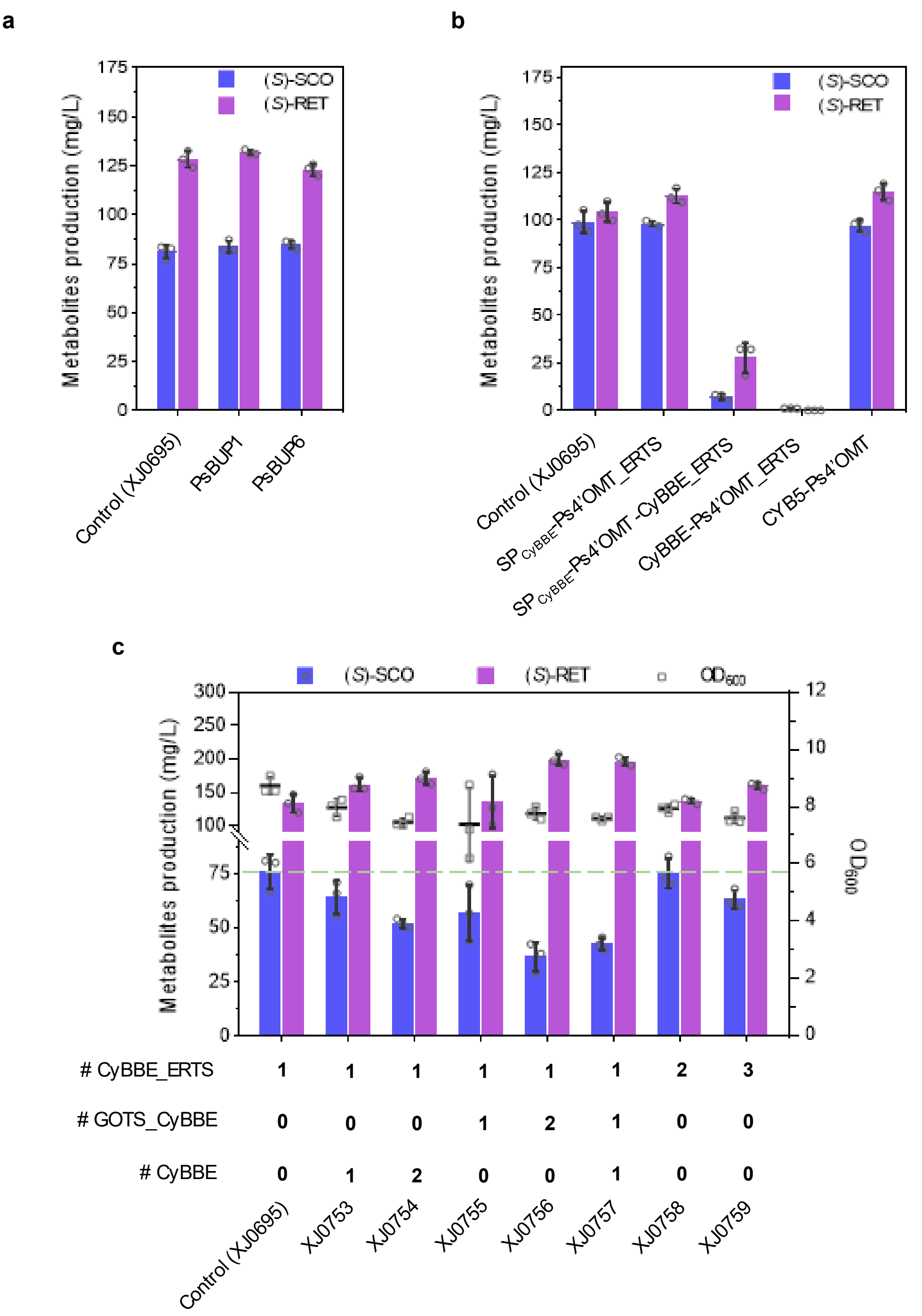

Supplementary Figure 18. (*S*)-SCO and (*S*)-RET titers in engineered strains. a either of purine permease-type BIA Transporters PsPUB1 and PsPUB6 was expressed, **b** Ps4'OMT was engineered to attach to ER membrane or target inside ER, and **c** extra copies of CyBBE_ERTS, GOTS_CyBBE or wild type CyBBE was introduced. *CYB5*, endogenous cytochrome b5 attaching to ER membrane. SP_{CyBBE} indicates N-terminal 29 amino acids of CyBBE. Data are presented as mean ± standard deviations (n = 3 biologically independent samples). Source data are provided as a Source Data file.

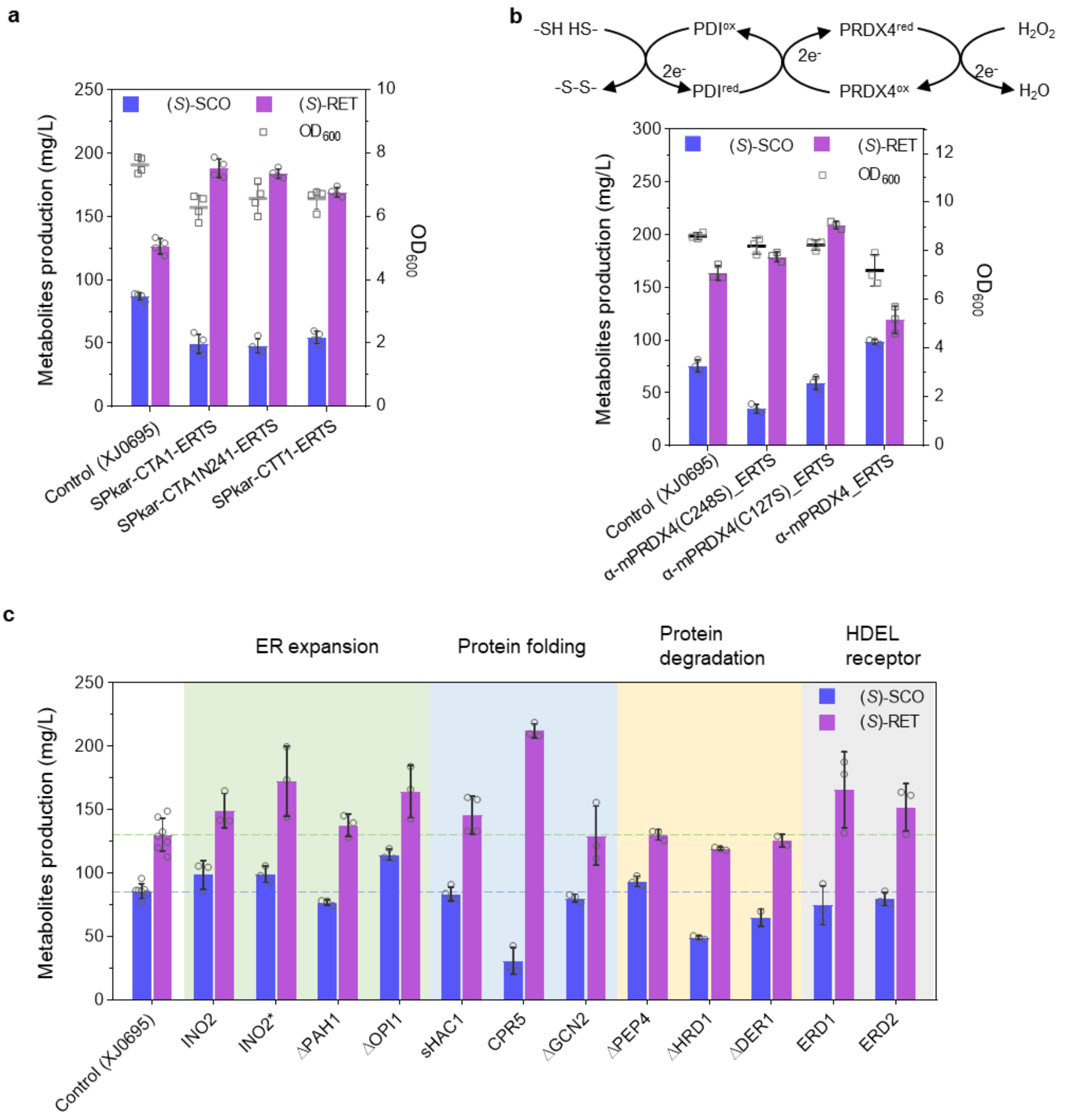

Supplementary Figure 19. (*S*)-SCO and (*S*)-RET titers in engineered strains. **a** endogenous catalases *CTT1*, *CTA1* and N-glycosylation-negative CTA1N241 were targeted inside ER, **b** mouse-derived PRDX4 and its nonsense mutations were expressed in ER, and **c** trafficking pathway was engineered. To test if ER-targeted catalases and PRDX4 could increase the (*S*)-SCO titer by H_2O_2 decomposition, these H_2O_2 decomposers were fused a N-terminal signal peptide (SP from *KAR2* for catalases and its variant, proalpha mating factor SP for PRDX4 and its mutations) and a C-terminal ERTS, respectively, and then transformed in control strain XJ0695. Modifications of trafficking pathway in yeast were performed in terms of ER expansion, protein folding, protein degradation and HDEL receptor overexpression. Data are presented as mean ± standard deviations (n = 3, 4 or 7 biologically independent samples). Source data are provided as a Source Data file.

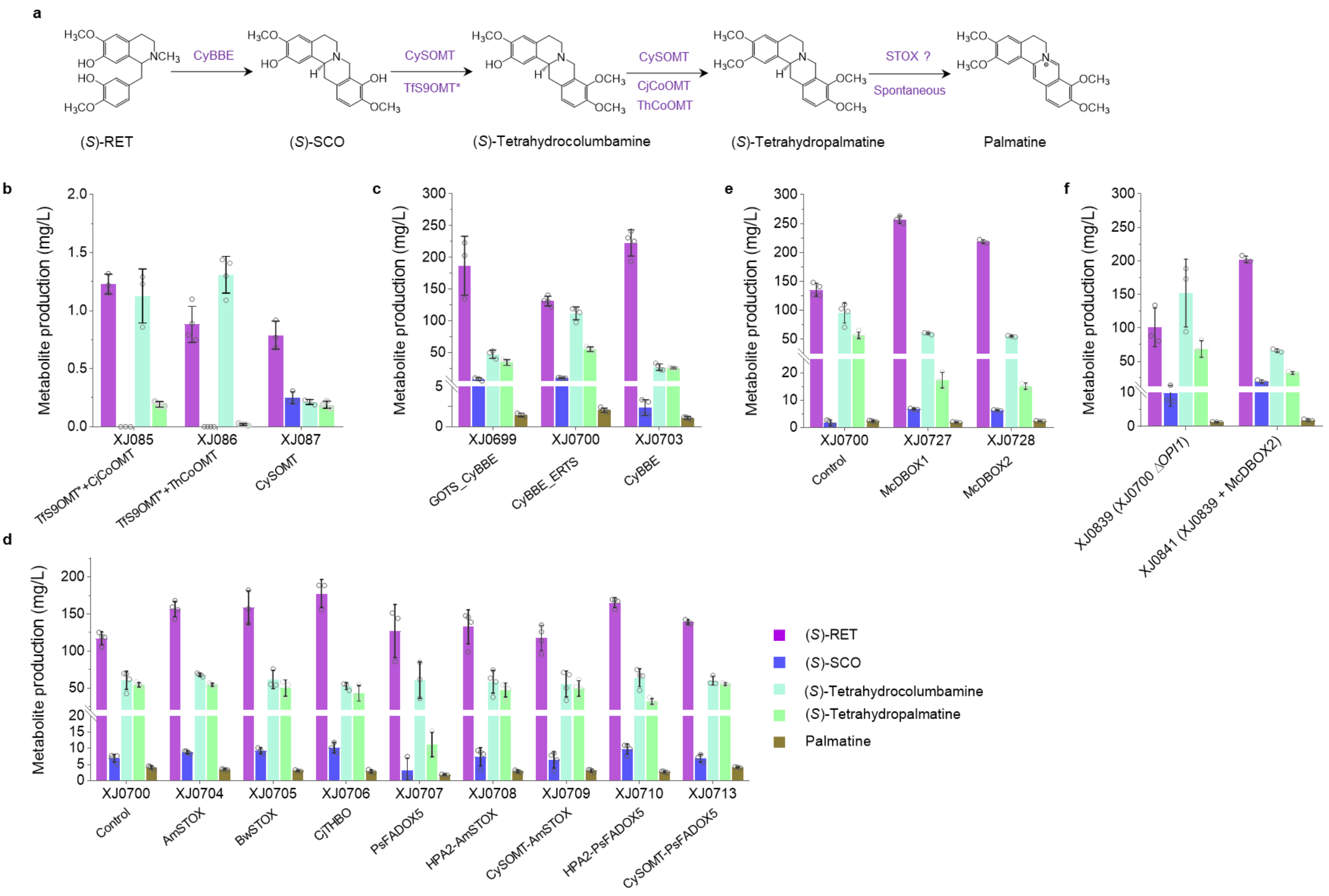

Supplementary Figure 20. Optimization of the module IV in yeast. a The biosynthetic pathway from (*S*)-RET to palmatine. **b** Selecting the optimal *O*-methyltransferases catalyzing (*S*)-SCO or (*S*)-tetrahydrocolumbamine. **c** Metabolites titers of module IV from engineered strains expressing TfS9OMT*, CySOMT, combined with wild type CyBBE, or its variants, GOTS_CyBBE and CyBBE_ERTS. **d** Screening STOX candidates catalyzing the final step from (*S*)-tetrahydropalmatine to palmatine. Besides AmSTOX, BwSTOX, CjTHBO and PsFADOX5, we constructed four fusions carrying N-terminal soluble domains displaying various oligomerization state, including HPA2-AmSTOX, CySOMT-AmSTOX, HPA2-PsFADOX5 and CysOMT-PsFADOX5. **e, f** Metabolites titer of module IV from engineered strains XJ0727, XJ0728 and XJ0841 expressing McDBOX1 or McDBOX2. Data are presented as mean ± standard deviations (n = 3 or 4 biologically independent samples). Source data are provided as a Source Data file.

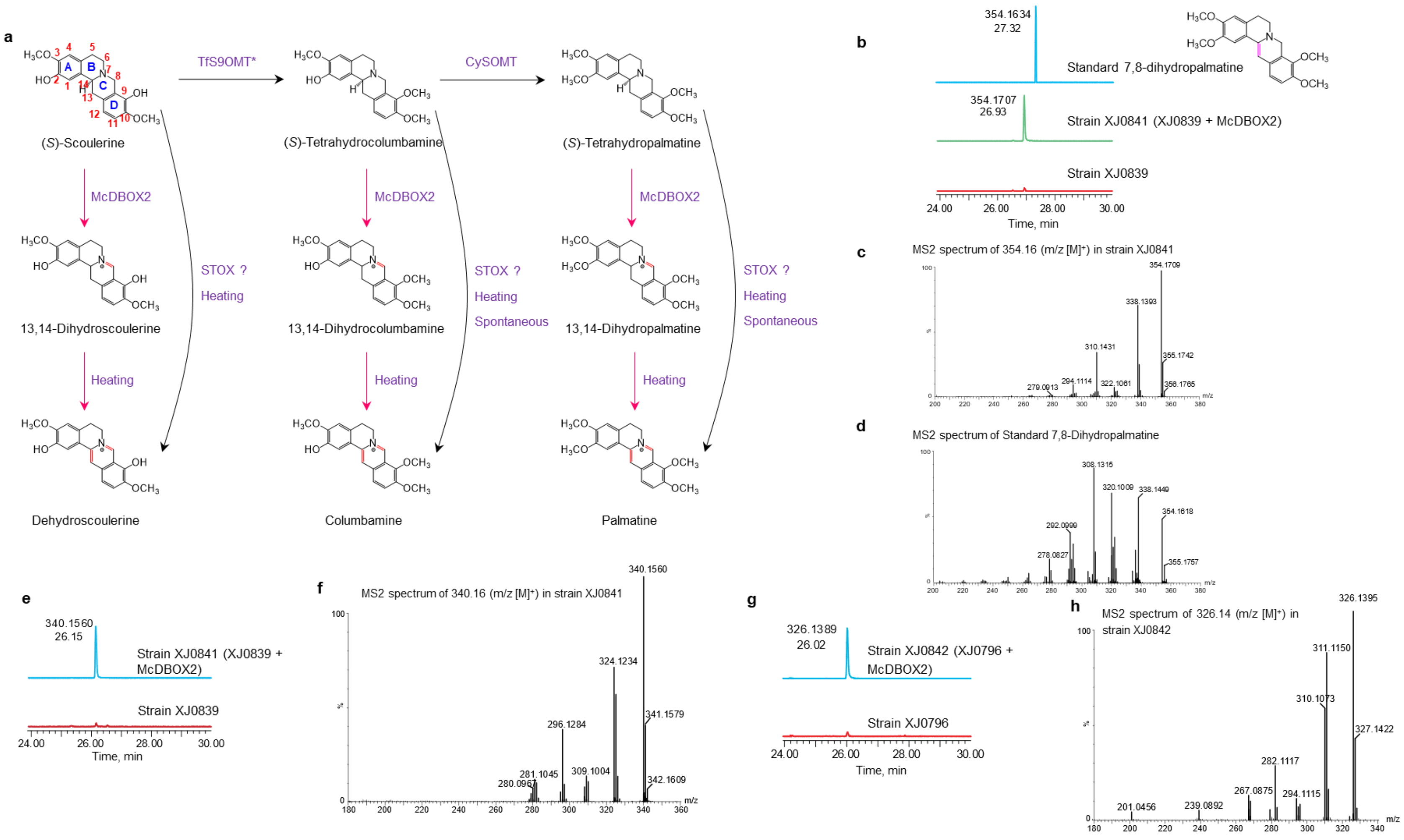

Supplementary Figure 21. McDBOX2 catalyzation on various tetrahydroprotoberberine substrates, (*S*)-SCO, (*S*)-tetrahydrocolumbamine and (*S*)-tetrahydropalmatine. a The oxidation pathway from tetrahydroprotoberberine to protoberberine. TIC of Q3 **b** (*m/z* 354.16 $[M]^+$) and **e** (*m/z* 340.16 $[M]^+$) in strain XJ0841 expressing McDBOX2. MS2 spectrum of **c** *m/z* 354.16 $[M]^+$ and **f** *m/z* 340.16 $[M]^+$ in strain XJ0841. **d** MS2 spectrum of commercial standard 7,8-dihydropalmatine. **g** TIC of Q3 and **h** MS2 spectrum of *m/z* 326.16 $[M]^+$ in strain XJ0842 expressing McDBOX2. Source data are provided as a Source Data file.

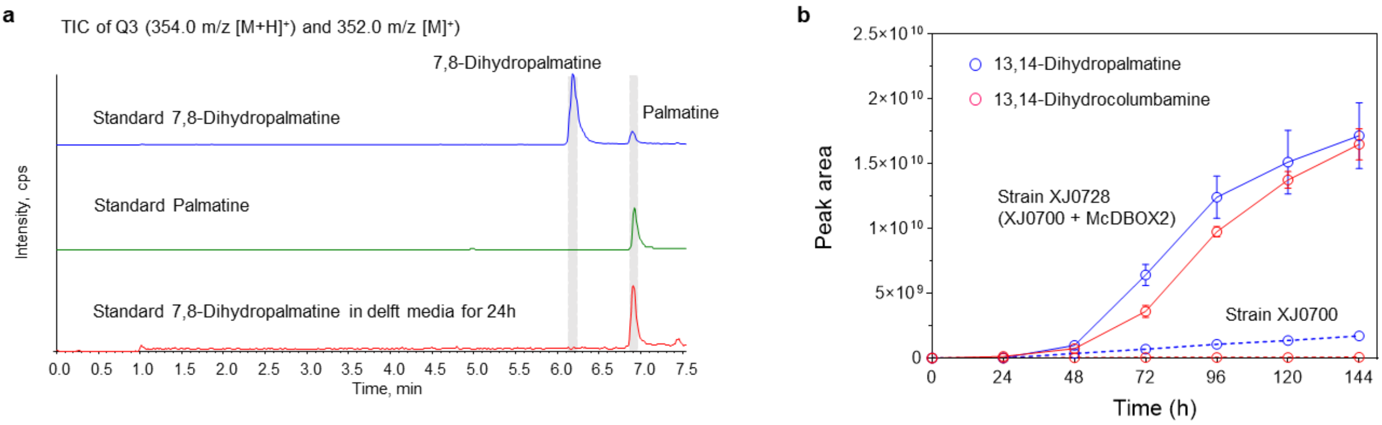

Supplementary Figure 22. The stabilization of commercial standard 7,8-dihydropalmatine, produced 13,14-dihydrocolumbamine and 13,14-dihydropalmatine from strain XJ0728. a TIC of Q3 (*m/z* 354.0 [M+H]$^+$ and 352.0 [M]$^+$) for detecting standard palmatine and 7,8-dihydropalmatine. **b** the production (peak area) of 13,14-dihydrocolumbamine and 13,14-dihydropalmatine from strain XJ0728 cultured for 144h. Three biologically independent colonies were grown in 20 mL delft media with 20 g/L glucose. Data are presented as mean ± standard deviations (n = 3 biologically independent samples). Source Data are provided as a Source Data file.

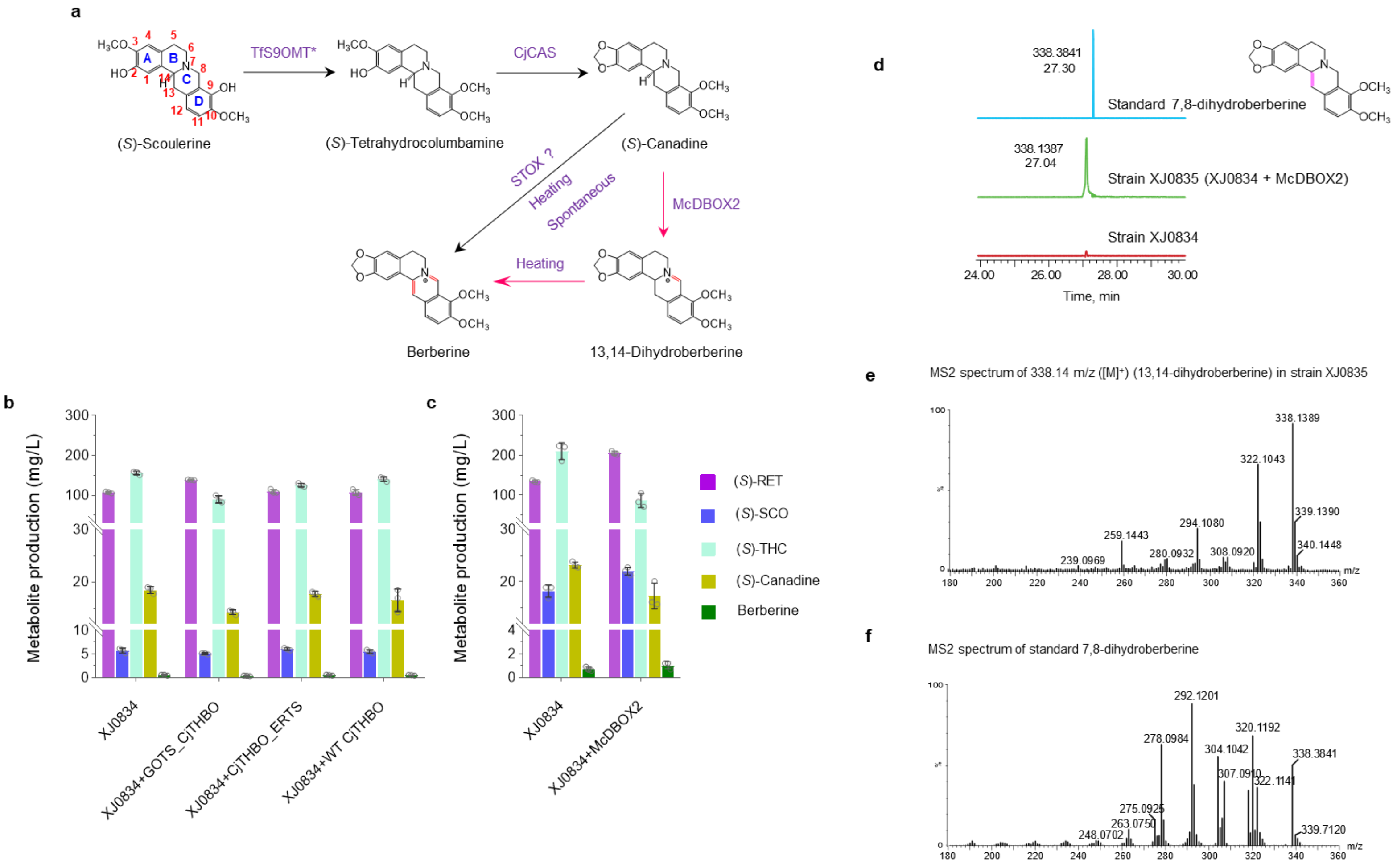

Supplementary Figure 23. Optimizing the module V in yeast. a The biosynthetic pathway from (*S*)-SCO to berberine. The metabolites production of module V from engineered strains expressing **b** CjTHBO, GOTS_CjTHBO, CjTHBO_ERTS and **c** McDBOX2. **d** TIC of Q3 and **e** MS2 spectrum of (*m/z* 338.14 $[M]^+$) in strain XJ0835 expressing McDBOX2. **f** MS2 spectrum of commercial standard 7,8-dihydroberberine. Data are presented as mean ± standard deviations (n = 3 biologically independent samples). Source data are provided as a Source Data file.

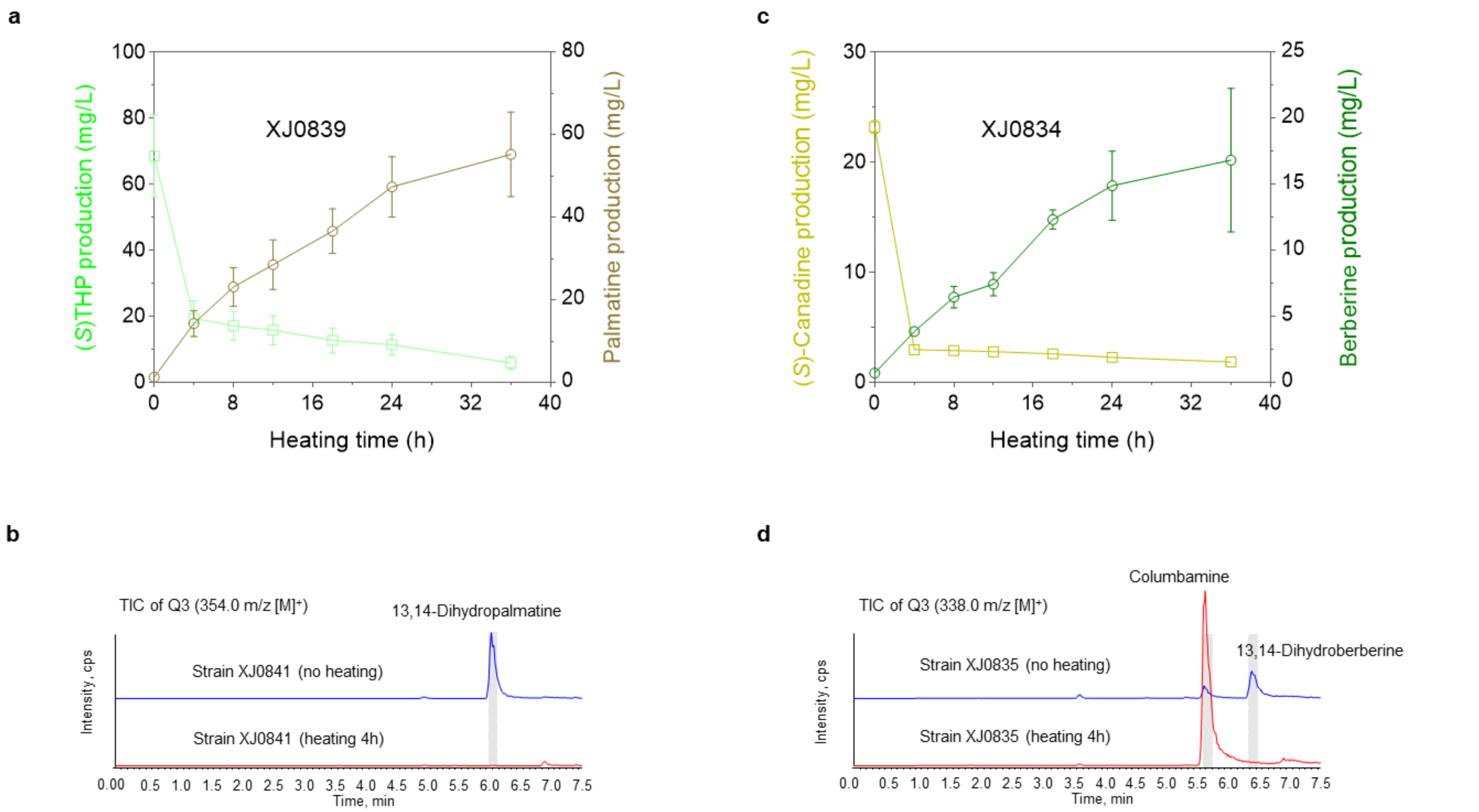

Supplementary Figure 24. The conversion of tetrahydroprotoberberines and dihydroprotoberberines to corresponding protoberberines under heating condition. a The production variation of palmatine and (*S*)-tetrahydropalmatine ((*S*)-THP) from the shake flask fermentation culture of strain XJ0839 followed by different heating time. **b** 13,14-dihydropalmatine was converted into palmatine by heating 4h. **c** The production variation of berberine and (*S*)-canadine from the shake flask fermentation culture of strain XJ0834 followed by different heating time. **d** 13,14-dihydroberberine was converted into berberine by heating 4h. Data are presented as mean ± standard deviations (n = 3 biologically independent samples). Source data are provided as a Source Data file.

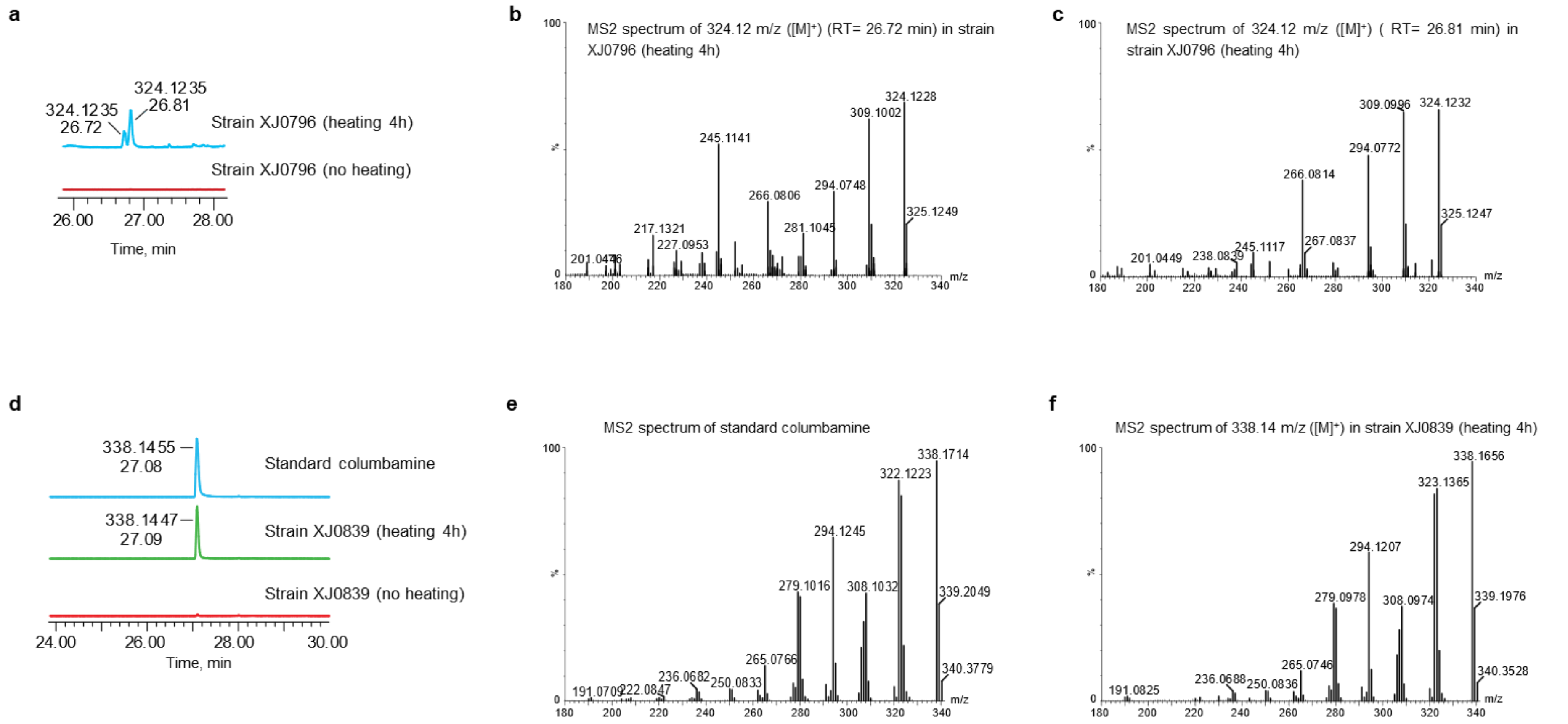

Supplementary Figure 25. Detection of additional two protoberberines, dehydroscoulerine and columbamine. a TIC of Q3 (*m/z* 324.12 $[M]^+$) from the culture of strains XJ0796 followed by heating 4h. MS2 spectrum of *m/z* 324.12 $[M]^+$ at **b** RT= 26.72 min and **c** RT= 26.81 min from strain XJ0796 followed by heating 4h. **d** TIC of Q3 (*m/z* 338.14 $[M]^+$) from the culture of strains XJ0839 followed by heating 4h. **e** MS2 spectrum of commercial standard columbamine. **f** MS2 spectrum of *m/z* 338.14 $[M]^+$ from strains XJ0839 followed by heating 4h. Source data are provided as a Source Data file.

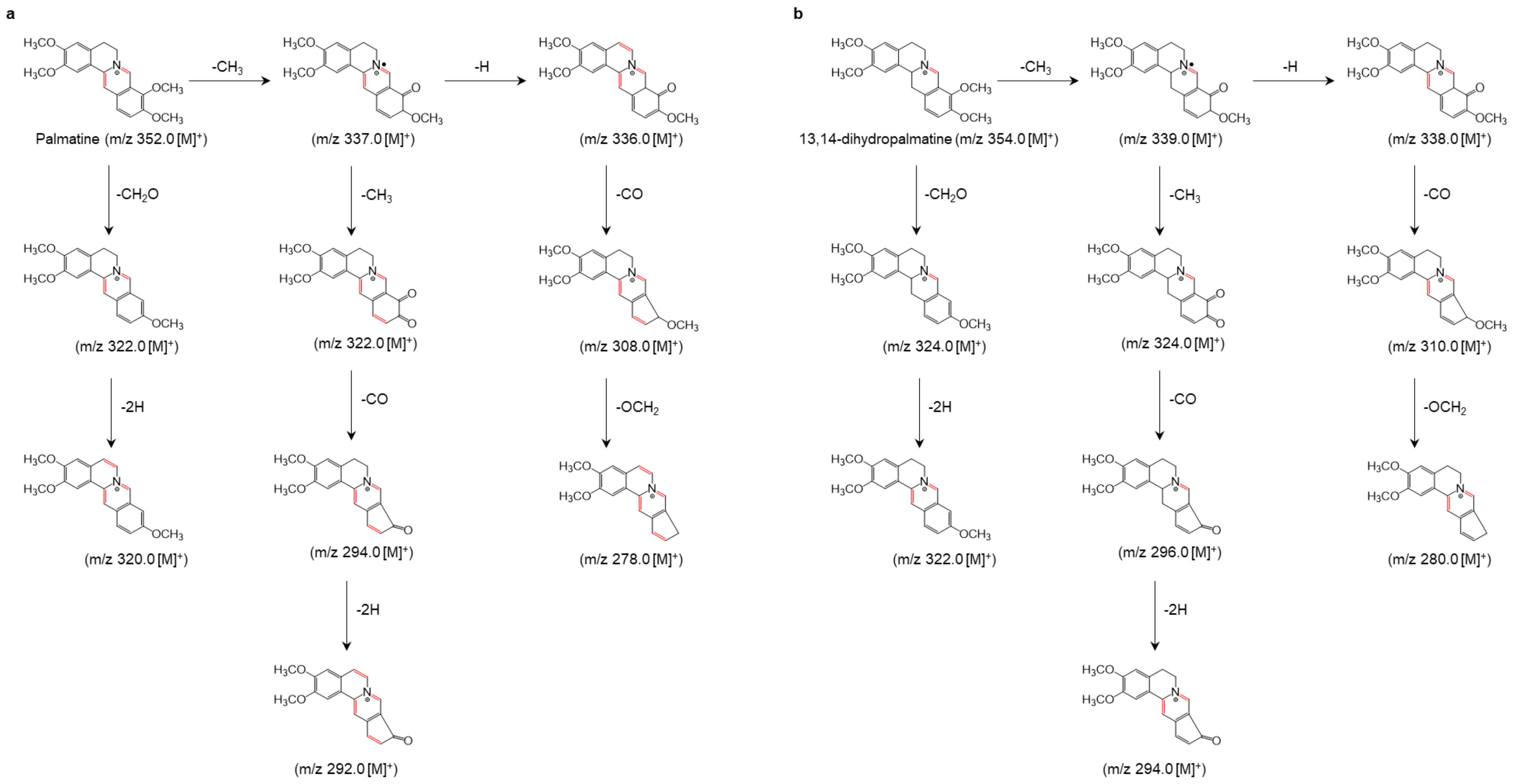

Supplementary Figure 26. MS2 spectrum and possible fragmentation pathway. a palmatine; **b** 13,14-dihydropalmatine.

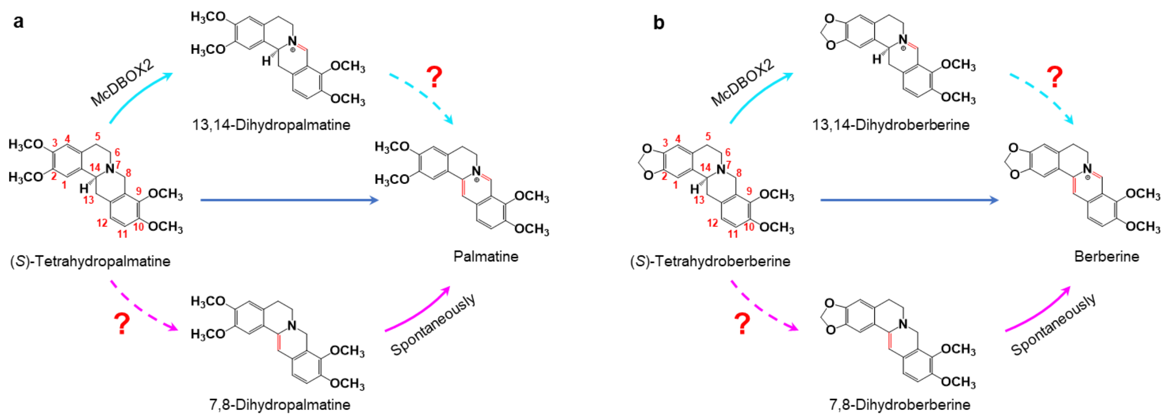

Supplementary Figure 27. The proposed pathways. a from (S)-tetrahydropalmatine to palmatine; **b** from (S)-tetrahydroberberine to berberine.

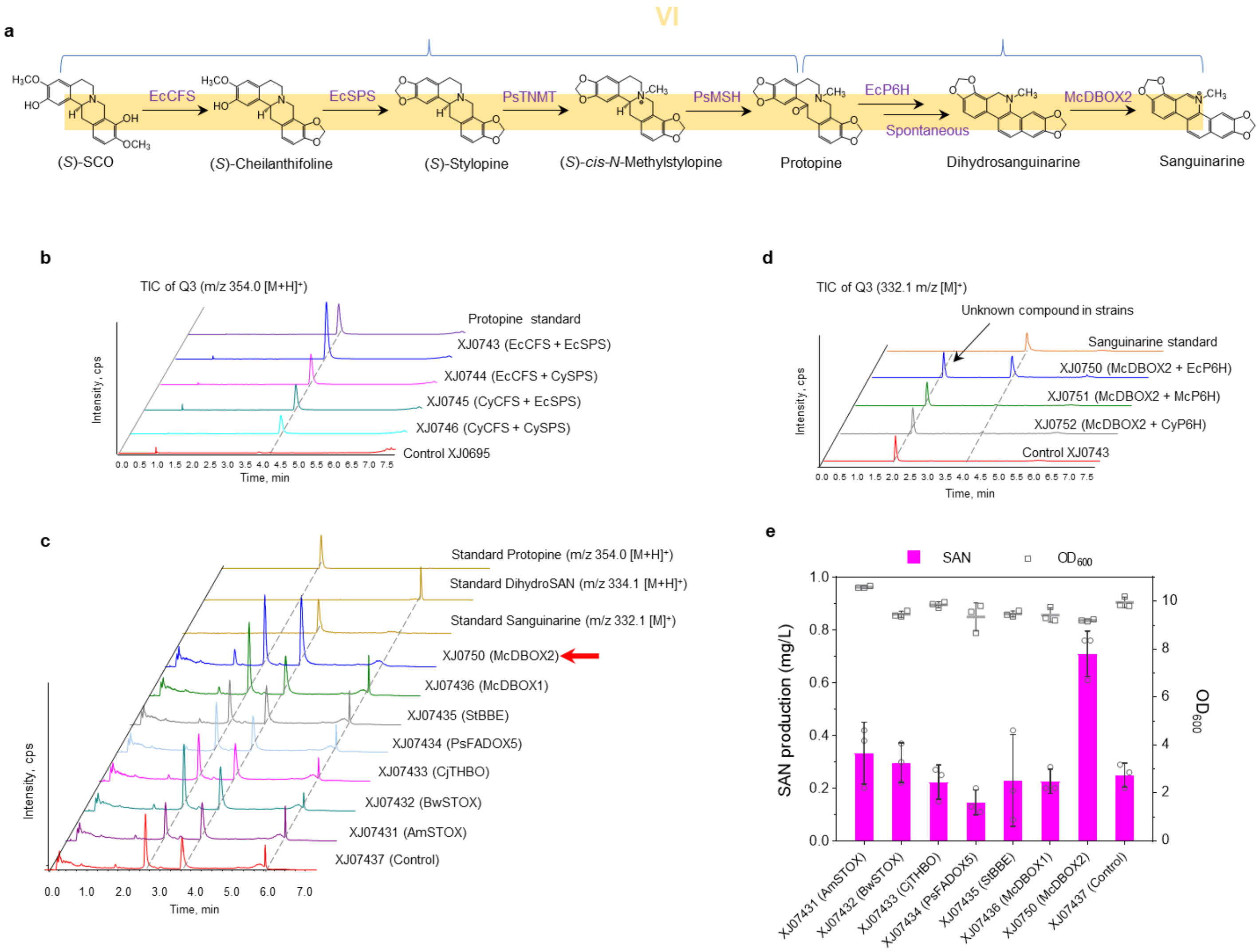

Supplementary Figure 28. Biosynthesize the intermediate protopine and sanguinarine (SAN) of module VI. a Schematic overview of module VI. **b** TIC of Q3 (*m/z* 354.0 $[M+H]^+$) in XJ0695-derived strains expressing distinctly originated CFS, SPS and PsTNMT, PsMSH. **c** TIC of Q3 (*m/z* 354.0 $[M+H]^+$, *m/z* 334.1 $[M+H]^+$ and *m/z* 332.1 $[M]^+$) and **e** SAN production and OD_{600} in engineered strains carrying different combinations of STOXs and EcP6H, respectively. Red arrow highlighted that McDBOX2 expressed in strain XJ0750 enabled conversion of dihydrosanguinarine to SAN. **d** TIC of Q3 (*m/z* 332.1 $[M]^+$) in engineered strains carrying different combinations of McDBOX2 and distinct P6Hs, respectively. Data are presented as mean ± standard deviations (n = 3 biologically independent samples). Source data are provided as a Source Data file.

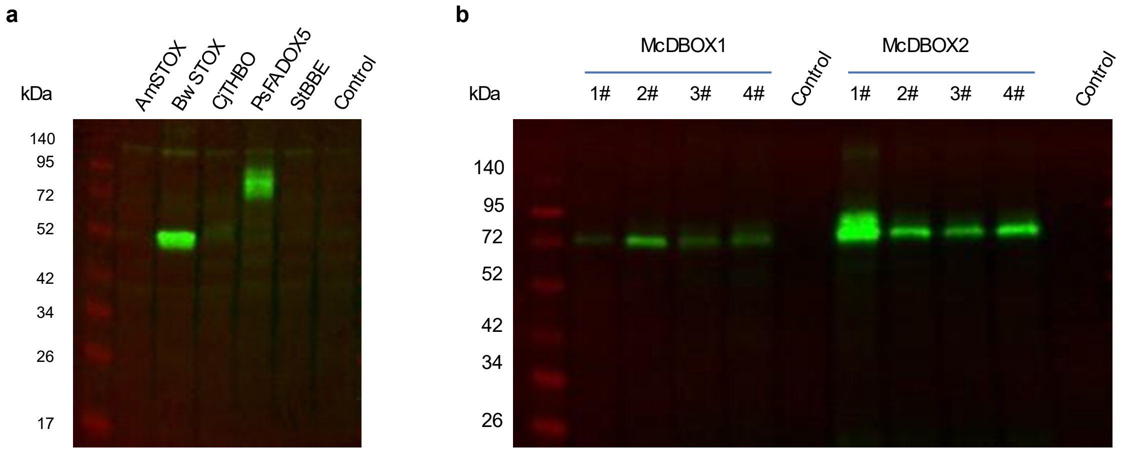

Supplementary Figure 29. The expression of various STOX candidates in yeast. Western blot analysis of **a** AmSTOX, BwSTOX, CjTHBO, PsFADOX5, StBBE, and **b** McDBOX1, McDBOX2. AmSTOX, BwSTOX, CjTHBO, PsFADOX5 and StBBE were assembled into plasmid P416_GPD, which were then transformed into wild type IMX581, which carrying P416 empty plasmid was acted as control. McDBOX1 and McDBOX2 were chromosomally expressed in strain XJ07436 and XJ0750, of which four biologically replicate colonies were randomly picked for western blot analysis, and XJ07437 as control. All candidates were fused with a C-terminal His-tag. Source data are provided as a Source Data file.

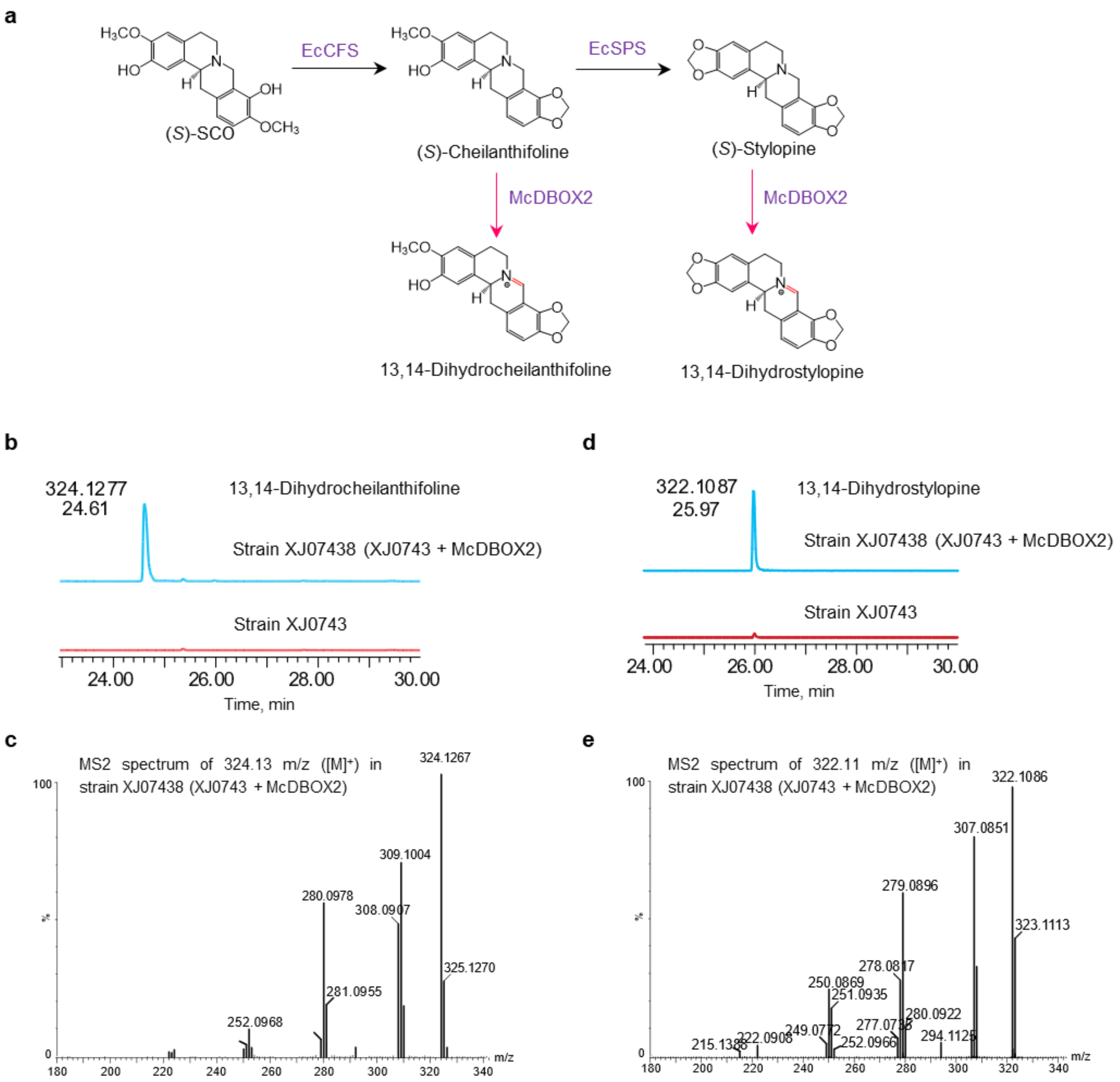

Supplementary Figure 30. McDBOX2 catalyzation on various tetrahydroprotoberberine substrates, (*S*)-cheilanthifoline and (*S*)-Stylopine. a The oxidation pathway from tetrahydroprotoberberine to protoberberine. TIC of Q3 **b** (*m/z* 324.13 $[M]^+$) and **d** (*m/z* 322.11 $[M]^+$) in strain XJ07438 expressing McDBOX2. MS2 spectrum of **c** *m/z* 324.13 $[M]^+$ and **e** *m/z* 322.11 $[M]^+$ in strain XJ07438 expressing McDBOX2. Source data are provided as a Source Data file.

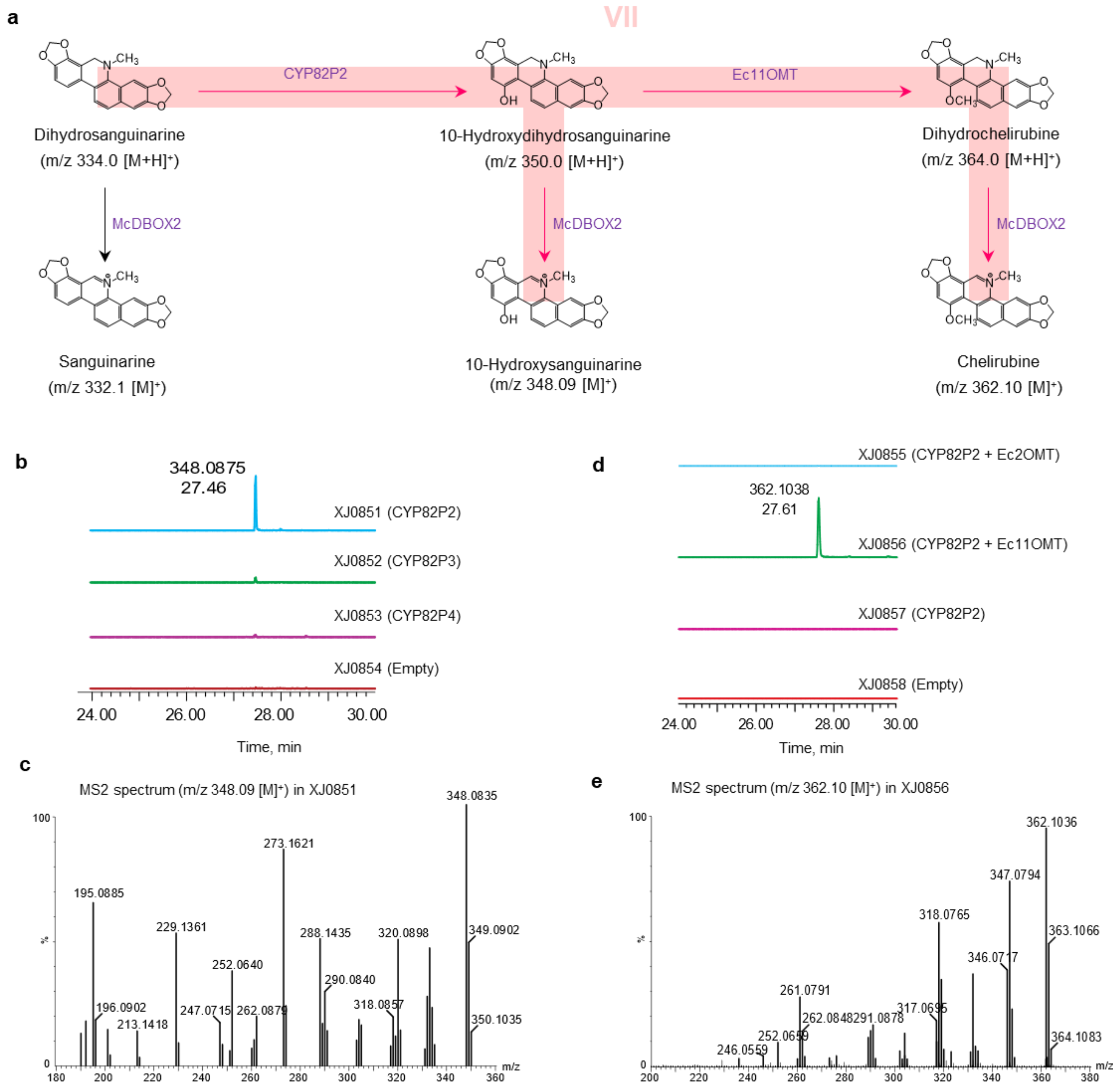

Supplementary Figure 31. Extending the pathway to biosynthesize Chelirubine. **a** Schematic presentation of Chelirubine biosynthetic pathway. CYP82P2, dihydrosanguinarine 10-hydroxylase from *E. californica*; Ec11OMT, 10-Hydroxydihydrosanguinarine 10-*O*-methyltransferase from *E. californica*; **b** TIC of Q3 (*m/z* 348.09 $[M]^+$) from engineered strains expressing plasmid-based three hydrolase candidates or empty plasmid, respectively. **c** MS2 spectrum of *m/z* 348.09 $[M]^+$ in strain XJ0851 expressing CYP82P2. **d** TIC of Q3 (*m/z* 362.10 $[M]^+$) from strains expressing plasmid-based CYP82P2 and two 10-*O*-methyltransferase candidates, single CYP82P2 or empty plasmid. **e** MS2 spectrum of *m/z* 362.10 $[M]^+$ in strain XJ0855 expressing CYP82P2 and Ec11OMT. Source data are provided as a Source Data file.

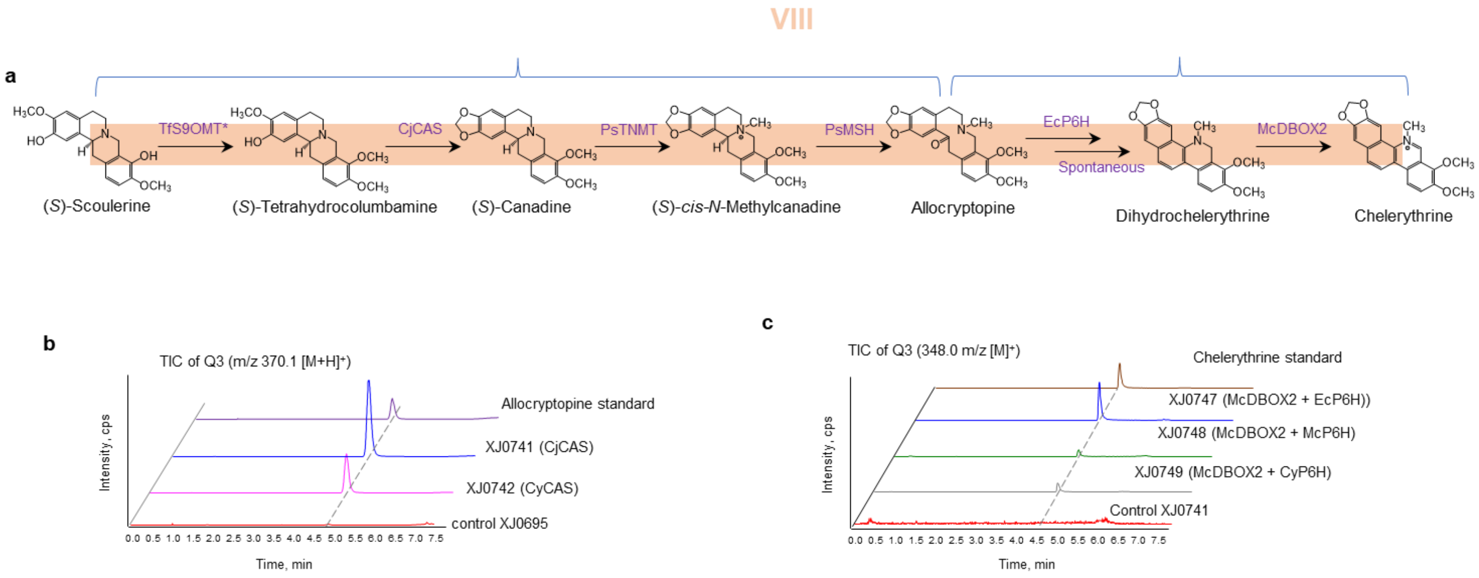

Supplementary Figure 32. Engineering the yeast to biosynthesize the intermediate allocryptopine and Chelerythrine of module VIII. a Schematic overview of module VIII. **b** TIC of Q3 (*m/z* 370.1 $[M+H]^+$) in XJ0695-derived strains expressing TfS9OMT*, PsTNMT, PsMSH and CjCAS (from *C. japonica*) or CyCAS (from *C. yanhusuo*). **c** TIC of Q3 (*m/z* 348.0 $[M]^+$) in engineered strains carrying different combinations of McDBOX2 and distinct P6Hs, respectively.

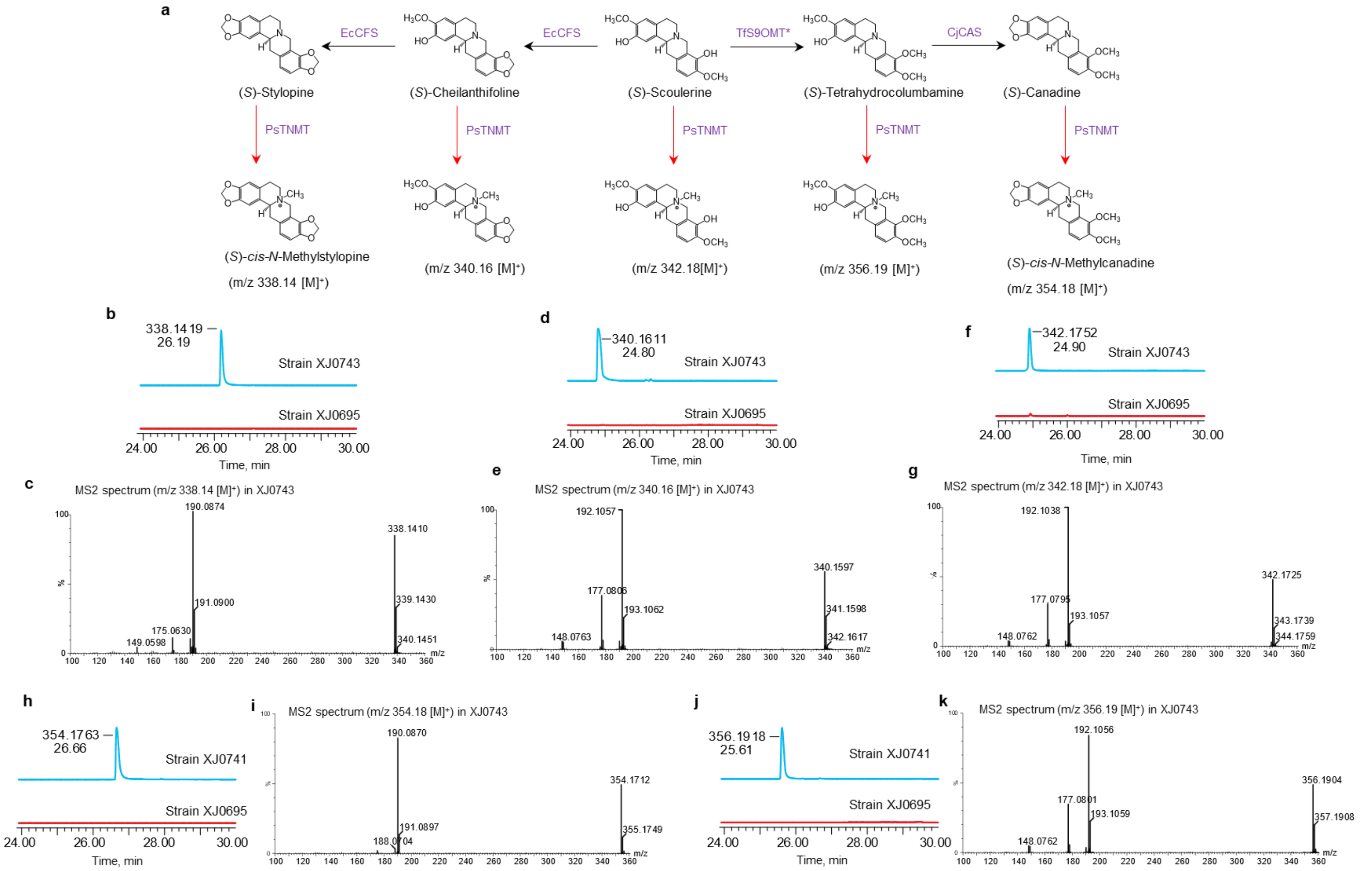

Supplementary Figure 33. PsTNMT promiscuity. a Pathway presentation of PsTNMT catalyzing distinct substrates. TIC of Q3 (**b** *m/z* 338.14 $[M]^+$, **d** *m/z* 340.16 $[M]^+$ and **f** *m/z* 342.18 $[M^+]$) in strain XJ0743. MS2 spectrum of **c** *m/z* 338.14 $[M]^+$, **e** *m/z* 340.16 $[M]^+$ and **g** *m/z* 342.18 $[M^+]$) in strain XJ0743. TIC of Q3 (**h** *m/z* 354.18 $[M]^+$ and **j** *m/z* 356.19 $[M^+]$) in strain XJ0741. MS2 spectrum of **i** *m/z* 354.18 $[M]^+$ and **k** *m/z* 356.19 $[M]^+$ in strain XJ0741.Source data are provided as a Source Data file.

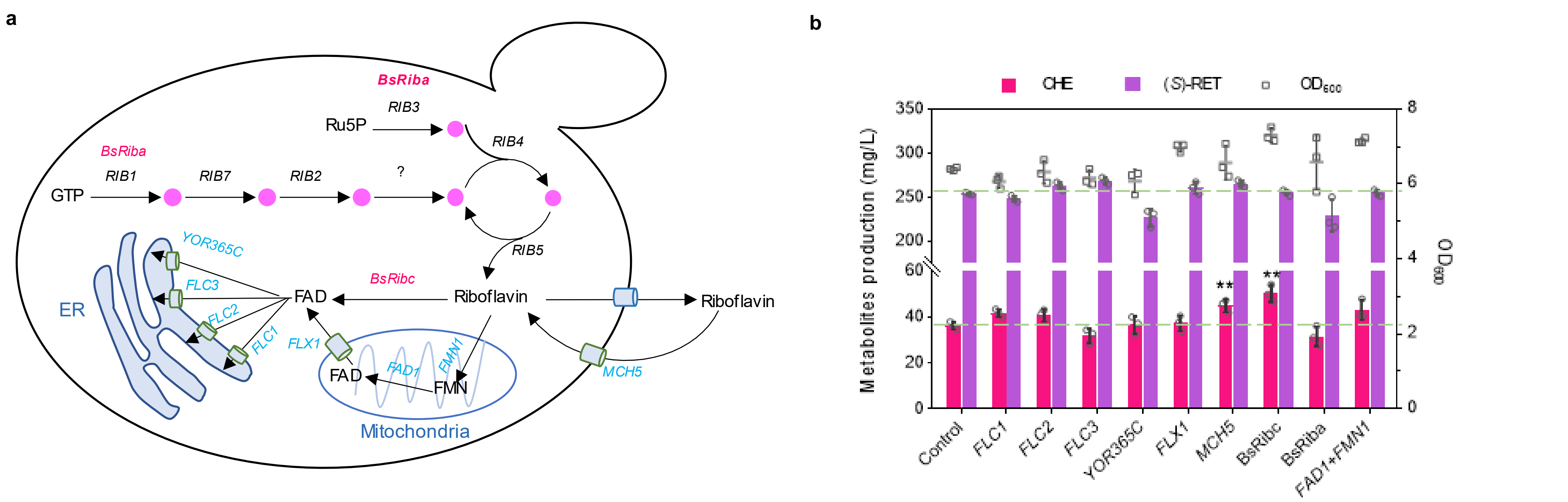

Supplementary Figure 34. Improving chelerythrine (CHE) titers by increasing the availability of cofactor FAD. a Schematic illustration of FAD biosynthesis and transport to ER in yeast. Blue indicates overexpression of endogenous genes, including four ER-attached FAD transporters *FLC1*, *FLC2*, *FLC3* and *YOR365C*, one mitochondria-localized transporter *FLX1*, one plasma membrane transporter *MCH5* and two FAD-biosynthesis-related genes *FAD1* and *FMN1*; pink indicates GTP cyclohydrolase II/ 3,4-dihydroxy-2-butanone 4-phosphate synthase BsRiba, and riboflavin kinase/ FAD synthase BsRibc, both from *B. subtilis*. **b** OD_{600}, CHE and (*S*)-RET titers in engineered strains expressing various transporters or FAD-biosynthesis-related enzymes. Data are presented as mean ± standard deviations (n = 3 biologically independent samples). Source data are provided as a Source Data file.

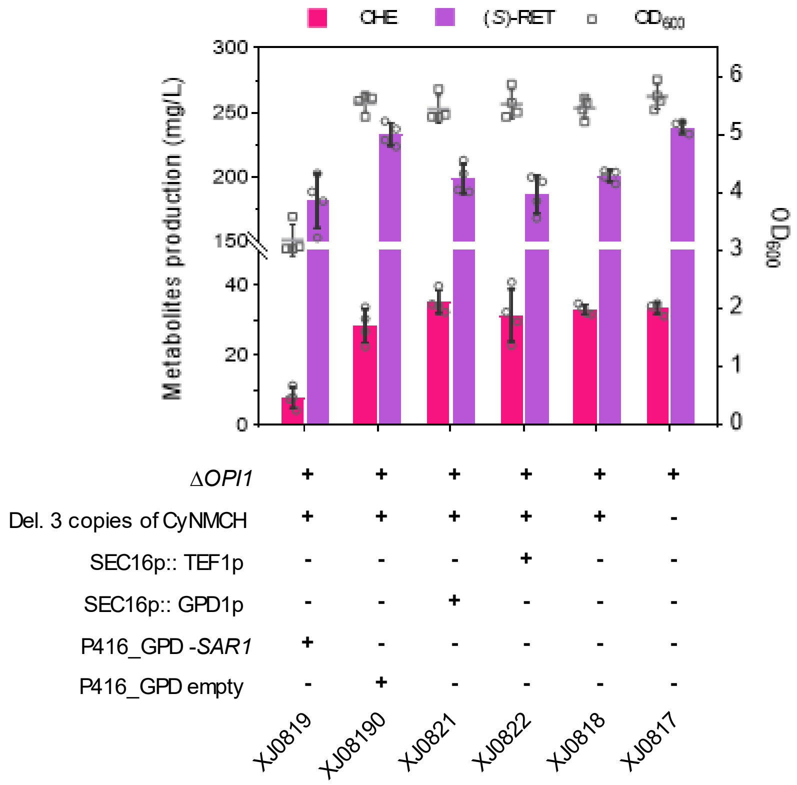

Supplementary Figure 35. CHE, (*S*)-RET titers and final OD_{600} in engineered strains. Engineering of COPII vesicle did not show significant improvements on CHE production, including plasmid-based overexpression of *SAR1* (encoding small GTPase component of COPII vesicles) or replacement of the promoter *SEC16* (involved in the initiation of assembly of COPII vesicles) with strong promoters GPD1p or TEF1p. Strain XJ0817 was acted as control, 3 copies of CyNMCH was deleted, aiming to alleviate the ER stress. Data are presented as mean ± standard deviations (n = 3 biologically independent samples). Source data are provided as a Source Data file.

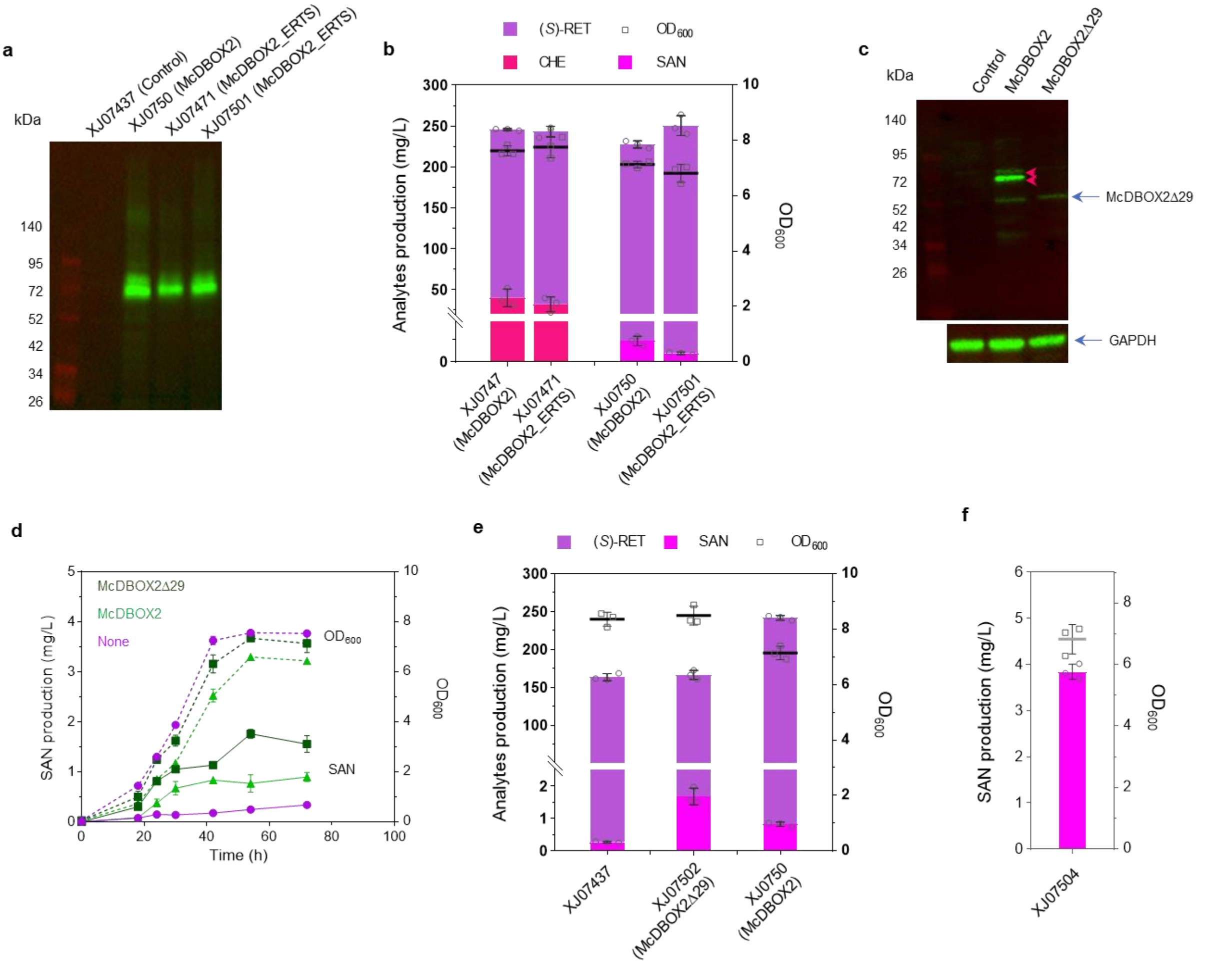

Supplementary Figure 36. Optimizing McDBOX2 expression in engineered yeast strains. a Western blot analysis of ER-localized McDBOX2 in strains XJ07471 and XJ07501 (XJ07437 as negative control; XJ0750 as positive control), and **c** McDBOX2Δ29 within p416 plasmid in IMX581 (IMX581 expressing plasmid p416_empty as negative control; IMX581 expressing plasmid p416_GPD-McDBOX2 as positive control). The theoretical protein sizes are 59.7 kDa (McDBOX2-His) and 56.8 kDa (McDBOX2Δ29-His). No matter McDBOX2 or McDBOX2_ERTS expressed, there are two bands, molecular weight corresponding to around 72 kDa, which might be caused by hyperglycosylation in yeast. McDBOX2Δ29 was cytosolically expressed, meaning that such truncation was not undergone post-transcriptional modifications, including hyperglycosylation, responsible for the missing two larger bands. **b** Metabolites titers and final OD_{600} in strains expressing McDBOX2 or McDBOX2_ERTS. **d** Time curve of SAN titer and OD_{600} in engineered strains expressing McDBOX2Δ29, McDBOX2 or no DBOX candidate. The solid line represents SAN production; the dashed line represents OD_{600}. Purple indicates the strain XJ07437 without the expression of DBOX candidate; light green indicates the strain XJ0750 (XJ07437 + McDBOX2) expressing wild type McDBOX2; dark green indicates the strain XJ07502 (XJ07437 + McDBOX2Δ29) expressing McDBOX2Δ29. **e** (*S*)-RET, sanguinarine (SAN) titers and OD_{600} in engineered strains XJ07437, XJ0750 (XJ07437 + McDBOX2) and XJ07502 (XJ07437 + McDBOX2Δ29). **f** SAN titer and final OD_{600} in final strain XJ07504 engineered with the improvement of FAD availability and *OPI1* deletion-mediated ER expansion. Data are presented as mean ± standard deviations (n = 3 biologically independent samples). Source data are provided as a Source Data file.

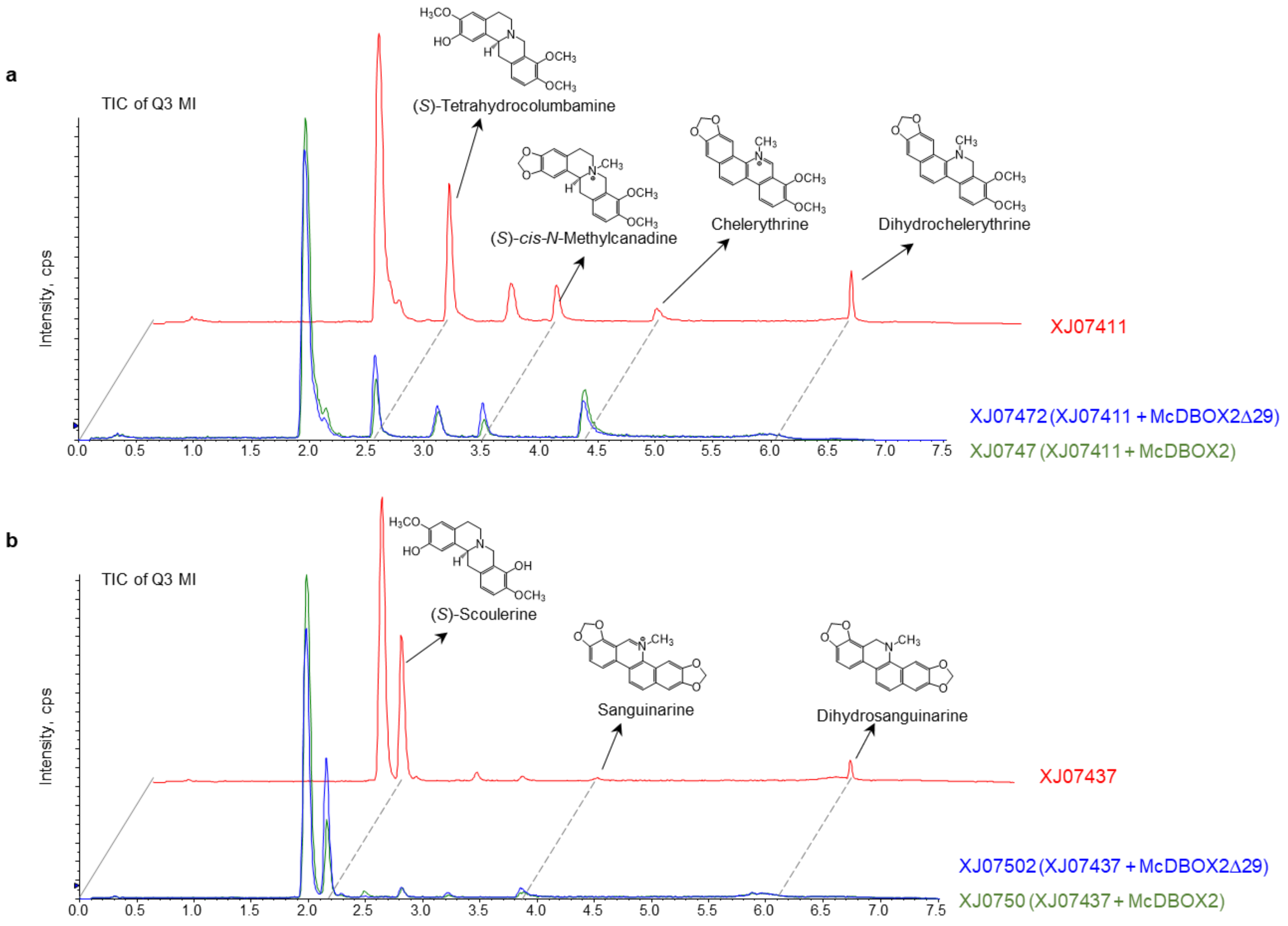

Supplementary Figure 37. TIC of Q3 MI. **a** TIC comparation of Q3 MI, including *m/z* corresponding to 330.0 $[M+H]^+$, 328.0 $[M+H]^+$, 342.0 $[M+H]^+$, 340.0 $[M+H]^+$, 354.0 $[M]^+$, 370.1 $[M+H]^+$, 350.0 $[M+H]^+$ and 348.0 $[M]^+$, respectively, in strains XJ07411, XJ07472 and XJ0747. **b** TIC comparation of Q3 MI, including *m/z* corresponding to 330.0 $[M+H]^+$, 328.0 $[M+H]^+$, 326.0 $[M+H]^+$, 324.0 $[M+H]^+$, 338.0 $[M]^+$, 354.0 $[M+H]^+$, 334.0 $[M+H]^+$ and 332.1 $[M]^+$, respectively, in strains XJ07437, XJ07502 and XJ0750.

Supplementary Table 1. Percentage of intracellular analytes titer out of whole titer.

Strain	Analyte	Q3 (*m/z* $[M+H]^+$) or MRM	Whole titer[1] (mg/L or peak area)	Intracellular titer[2] (mg/L or peak area)	Percentage (% intracellular titer/ whole titer)
XJ0827	(*S*)-RET	330.1-137.2	284.1 ± 24.1	13.4 ± 0.8	4.7
	(*S*)-SCO	328.2-163.2	*[3]	*	-
	(*S*)-Tetrahydrocolumbamine	342.2-163.1	24.5 ± 3.1	1.4 ± 0.3	5.7
	(*S*)-Tetrahydroberberine	340.0	*	*	-
	(*S*)-*cis*-*N*-Methylcanadine	354.0	2.55E+09 ± 3.75E+08	9.16E+07 ± 3.66E+07	3.6
	Allocryptopine	370.0	1.19E+10 ± 1.50E+08	4.79E+08 ± 4.52E+07	4.0
	Dihydrochelerythrine	350.1	*	*	-
	Chelerythrine	348.0-332.1	66.9 ± 7.9	6.4 ± 1.3	9.6
XJ0832	(*S*)-RET	330.1-137.2	84.8 ± 3.4	3.7 ± 0.9	4.4
	(*S*)-SCO	328.2-163.2	13.0 ± 0.2	0.6 ± 0.2	4.6
	(*S*)-Cheilanthifoline	326.0	*	*	-
	(*S*)-Stylopine	324.0	*	*	-
	(*S*)-*cis*-*N*-Methylstylopine	338.0	2.79E+09 ± 9.92E+07	1.29E+08 ± 2.98E+07	4.6
	Protopine	354.0	7.36E+08 ± 2.11E+07	3.73E+07 ± 8.69E+06	5.1
	Dihydrosanguinarine	334.0	*	*	-
	Sanguinarine	332.0-316.9	5.0 ± 0.1	1.1 ± 0.0	22

[1] whole titer was obtained by sample extraction from culture.
[2] intracellular titer was obtained from cell pellet.
[3] lower than detection limitation.
Source data are provided as a Source Data file.

Supplementary Table 2. Paramators of LC-MS/MS for the detection of target compounds.

**Compound**	**MRM mode for quantification or qualification**	**DP (V)**	**EP (V)**	**CE (V)**	**CXP (V)**
Dopamine	154.0-118.9	1	10	25	46
(*S*)-NOR	271.8-106.9	26	10	33	52
(*S*)-RET	329.9-137.2	91	10	37	10
4-HPAC	151.0-107.0	-45	-10	-22	-13
Tyrosol	136.9-106.0	-40	-10	-20	-13
(*S*)-Coclaurine	285.1-107.0	40	10	30	25
(*S*)-*N*-Methylcoclaurine	299.2-107.0	40	10	30	25
(*S*)-3'Hydroxy-*N*-Methylcoclaurine	315.1-192.0	40	10	30	25
(*S*)-SCO	328.2-163.2	50	9	47	14
(*S*)-Tetrahydrocolumbamine	342.2-163.1	70	5	59	14
Chelerythrine	348.0-332.1	70	7	39	38
Sanguinarine	332.0-316.9	60	5	43	44
(*S*)-Tetrahydropalmatine	356.0-175.9	51	10	67	18
Palmatine	352.1-335.9	31	10	41	46
(*S*)-Canadine	340.1-149.1	40	10	37	18
Berberine	336.1-320.0	40	10	41	38
Q3 mode for qualification					
(*S*)-Tetrahydroberberine	340.0	130	10	-	40
(*S*)-*cis*-*N*-Methylcanadine	355.0	130	10	-	40
Allocryptopine	370.0	130	10	-	40
Dihydrochelerythrine	350.1	130	10	-	40
(*S*)-Cheilanthifoline	326.0	130	10	-	40
(*S*)-Stylopine	324.0	130	10	-	40
(*S*)-*cis*-*N*-Methylstylopine	339.0	130	10	-	40
Protopine	354.0	130	10	-	40
Dihydrosanguinarine	334.0	130	10	-	40
MS2 mode for qualification					
Allocryptopine	370.0	130	10	15-45	17
Chelerythrine	348.0	130	10	15-45	17
Protopine	354.0	130	10	15-45	17
Sanguinarine	332.1	130	10	15-45	17
10-hydroxysanguinarine	348.0	130	10	15-45	17
Chelirubine	364.0	130	10	15-45	17
13,14-dihydroscoulerine	326.0	130	10	15-45	17
Dehydroscoulerine	324.0	130	10	15-45	17
13,14-dihydrocolumbamine	340.0	130	10	15-45	17
Columbamine	338.0	130	10	15-45	17
13,14-dihydropalmatine	354.0	130	10	15-45	17
13,14-dihydroberberine	338.0	130	10	15-45	17

Supplementary references

1. Nishihachijo, M. et al. Asymmetric synthesis of tetrahydroisoquinolines by enzymatic Pictet–Spengler reaction. *Bioscience, Biotechnology, and Biochemistry* **78**, 701-707 (2014).
2. Fischbach, A. et al. Artificial Hsp104-mediated systems for re-localizing protein aggregates. *Nat Commun* **14**, 2663 (2023).
3. Lindquist, S.K.a.S. Aggregation of huntingtin in yeast varies with thelength of the polyglutamine expansion and theexpression of chaperone proteins. *PNAS* **97**, 1589 –1594 (2000).
4. Petroi, D. et al. Aggregate clearance of alpha-synuclein in Saccharomyces cerevisiae depends more on autophagosome and vacuole function than on the proteasome. *J Biol Chem* **287**, 27567-27579 (2012).
5. Orij, R., Brul, S. & Smits, G.J. Intracellular pH is a tightly controlled signal in yeast. *Biochim Biophys Acta* **1810**, 933-944 (2011).
6. McIlvaine, T.C. A buffer solution for colorimetric comparison. *J. Biol. Chem.* **49**, 183-186 (1921).
7. Paul Steffens, N.N., Meinhart H. Zenk Purification and characterization of the berberine bridge enzyme from berberis beaniana cell cultures. *Phytochemistry* **24**, 2577-2583 (1985).
8. Winkler, A. et al. A concerted mechanism for berberine bridge enzyme. *Nat Chem Biol* **4**, 739-741 (2008).
9. Usera, A.R. & O'Connor, S.E. Mechanistic advances in plant natural product enzymes. *Curr Opin Chem Biol* **13**, 492-498 (2009).
10. Gaweska, H.M., Roberts, K.M. & Fitzpatrick, P.F. Isotope effects suggest a stepwise mechanism for berberine bridge enzyme. *Biochemistry* **51**, 7342-7347 (2012).
11. Winkler, A. et al. Berberine bridge enzyme catalyzes the six electron oxidation of (*S*)-reticuline to dehydroscoulerine. *Phytochemistry* **70**, 1092-1097 (2009).
12. Liu, L. et al. Engineering the biosynthesis of caffeic acid in *Saccharomyces cerevisiae* with heterologous enzyme combinations. *Engineering* **5**, 287-295 (2019).
13. Kodama, Y. & Hu, C.D. An improved bimolecular fluorescence complementation assay with a high signal-to-noise ratio. *Biotechniques* **49**, 793-805 (2010).
14. Schenck, C.A. & Maeda, H.A. Tyrosine biosynthesis, metabolism, and catabolism in plants. *Phytochemistry* **149**, 82-102 (2018).
15. Schmidt, J., Raith, K., Boettcher, C. & Zenk, M.H. Analysis of benzylisoquinoline-type alkaloids by electrospray tandem mass spectrometry and atmospheric pressure photoionization. *European Journal of Mass Spectrometry* **11**, 325-333 (2005).
16. Bird, D.A. & Facchini, P.J. Berberine bridge enzyme, a key branch-point enzyme in benzylisoquinoline alkaloid biosynthesis, contains a vacuolar sorting determinant. *Planta* **213**, 888-897 (2001).
