# Supplementary material for: De novo production of protoberberine and benzophenanthridine alkaloids through metabolic engineering of yeast
Source: Nat Commun. 2024 Oct 9;15:8759. doi: 10.1038/s41467-024-53045-3 (PMC11464499; doi:10.1038/s41467-024-53045-3)
Supplement: Supplementary file 3 — Description of Additional Supplementary Files [file 41467_2024_53045_MOESM3_ESM.pdf]

Description of Additional Supplementary Files

Supplementary Data 1. Strains used in this paper

Supplementary Data 2. Primers used in this paper

Supplementary Data 3. Gene sequence used in this paper

Supplementary Data 4. Plasmids used in this paper
